# Development of the serotonergic cells in murine raphe nuclei and their relations with rhombomeric domains

**DOI:** 10.1007/s00429-012-0456-8

**Published:** 2012-09-30

**Authors:** Antonia Alonso, Paloma Merchán, Juan E. Sandoval, Luisa Sánchez-Arrones, Angels Garcia-Cazorla, Rafael Artuch, José L. Ferrán, Margaret Martínez-de-la-Torre, Luis Puelles

**Affiliations:** 1 Department of Human Anatomy and Psychobiology, Faculty of Medicine, School of Medicine, University of Murcia, 30071 Murcia, Spain; 2 Division of Developmental Biology, Cincinnati Children’s Hospital Medical Center, University of Cincinnati College of Medicine, 3333 Burnet Avenue, Cincinnati, OH 45229 USA; 3 Institute du Thorax, Université de Nantes, UMR, 1087 Nantes, France; 4 Centro de Biología Molecular Severo Ochoa, CSIC-Universidad Autónoma de Madrid, Cantoblanco, 28049 Madrid, Spain; 5 Neuropediatrics and Clinical Biochemistry Departments, Hospital Sant Joan de Déu, Barcelona, Spain; 6 Biomedical Network of Research Centres on Rare Diseases (CIBER-ER), Instituto de Salud Carlos III, Madrid, Spain

**Keywords:** Hindbrain, Rhombomeres, Serotonin, Midbrain, Pet1, Lmx1b

## Abstract

The raphe nuclei represent the origin of central serotonergic projections. The literature distinguishes seven nuclei grouped into rostral and caudal clusters relative to the pons. The boundaries of these nuclei have not been defined precisely enough, particularly with regard to developmental units, notably hindbrain rhombomeres. We hold that a developmental point of view considering rhombomeres may explain observed differences in connectivity and function. There are twelve rhombomeres characterized by particular genetic profiles, and each develops between one and four distinct serotonergic populations. We have studied the distribution of the conventional seven raphe nuclei among these twelve units. To this aim, we correlated 5-HT-immunoreacted neurons with rhombomeric boundary landmarks in sagittal mouse brain sections at different developmental stages. Furthermore, we performed a partial genoarchitectonic analysis of the developing raphe nuclei, mapping all known serotonergic differentiation markers, and compared these results, jointly with others found in the literature, with our map of serotonin-containing populations, in order to examine regional variations in correspondence. Examples of regionally selective gene patterns were identified. As a result, we produced a rhombomeric classification of some 45 serotonergic populations, and suggested a corresponding modified terminology. Only a minor rostral part of the dorsal raphe nucleus lies in the midbrain. Some serotonergic neurons were found in rhombomere 4, contrary to the conventional assumption that it lacks such neurons. We expect that our reclassification of raphe nuclei may be useful for causal analysis of their differential molecular specification, as well as for studies of differential connectivity and function.

## Introduction

Serotonergic neurons associated to raphe nuclei are represented throughout the hindbrain and nowhere else in the brain (with the exception of minor midbrain and spinal additions). Nieuwenhuys (1985) reviewed literature showing that this cell type normally coexists in these nuclei with other sorts of neurons, in variable proportions. In fact, a retropontine ‘nucleus raphe interpositus’ is mentioned in the literature that holds no serotonergic neurons at all (Büttner-Enever et al. 1998). In the present work we concentrate on the serotonergic populations.

We now know that the hindbrain is organized developmentally in a series of transverse neuromeric units, generically named rhombomeres (Puelles et al. 2007; Nieuwenhuys et al. 2008; Nieuwenhuys 2011; Watson and Paxinos 2010). It can be deduced from known descriptions that each rhombomere probably produces a specific part of the series of raphe nuclei, but the corresponding distribution has not been determined yet. Recently, Jensen et al. (2008) used triple transgenic mappings to locate serotonergic populations derived, respectively, from r1, r2, r3 and r5, concluding that r1–r3 contribute more or less discretely to the classic rostral raphe nuclei (see “Discussion”). This issue is of interest, at least in order to understand the specificities observed in the projection targets and consequent possible function and pathophysiology of the individual raphe nuclei. This implies that observed differential properties derive from the singular molecular identity of their respective neuromeric origins.

Raphe nuclei were subdivided classically into rostral and caudal clusters, and given specific names (Taber et al. 1960; Lidov and Molliver 1982; Aitken and Törk 1988; Table 1). According to Dahlström and Fuxe (1964), the classic raphe nuclei fall into the following alphanumeric classification: the rostral cluster is represented by principal and caudal subdivisions of the *dorsal raphe nucleus* (DR/B7 and cDR/B6), jointly with the *caudal linear nucleus* and the *median raphe nucleus,* also known as ‘central superior raphe nucleus’ (CLi + MnR/B8), the *supralemniscal raphe nucleus* (SuL/B9) and the *pontine raphe nucleus* (PnR/B5). The classical caudal cluster is formed by the following major groups: *supragenual nucleus* (SGeR/B4), *nucleus raphe magnus* (RMg/B3), *nucleus raphe obscurus* (ROb/B2) and *nucleus raphe pallidus* (RPa/B1), to which a group of *parapyramidal* serotonergic neurons can be added.

**Table 1:** Names and abbreviations proposed for raphe nuclei in this work, compared to classical and alpha-numeric terms

Traditional terminology and classification	Alpha-numeric terminology (Dahlström and Fuxe 1964; Seiger and Olson 1973)	Presently proposed terminology and abbreviations	Other denominations
Dorsal raphe n. (DR) (Ramón y Cajal 1909; Brown 1943; Olzewski and Baxter 1954; Taber et al. 1960; Steinbuch and Nieuwenhuys 1985; Jacobs and Azmitia 1992; Paxinos and Franklin 2007; Paxinos and Watson 2007)	B7	Dorsal raphe nn. mDR	n. supratrochlearis (Olszewski and Baxter 1954)
		isDRd isDRl isDRv isDRW	DRD/DRV/DRW-DRVL/DRL (Steinbusch 1981; Diaz-Cintra et al. 1981; Agnati et al. 1982; Paxinos and Franklin 2007; Abrams et al. 2004)
	B6	r1DRd r1DRv r1DRW	
Caudal linear n. (CLi) (Castaldi 1923; Steinbuch and Nieuwenhuys 1985; Jacobs and Azmitia 1992)	B8	Caudal linear n. (Is) CLi	Linear intermediate n. (Brown 1943; Taber et al. 1960)
		Caudal linear wing n. (Is) CLiW	n. pontis oralis, pars rostralis (Jacobs et al. 1984; Azmitia and Gannon 1986; Hornung and Fritschy 1988; Törk and Hornung 1990)
Central superior raphe n. (CS) (Bechterew 1899; Olszewski and Baxter 1954; Taber et al. 1960; Valverde 1962)		Median raphe nn. r1Mnr r1Mnc	Median raphe n. (Jacobs and Azmitia 1992; Paxinos and Franklin 2007; Paxinos and Watson 2007)
			n. linearis caudalis (Brown 1943)
		Prepontine raphe nn. (r2) PPnR	Caudal portion of the central superior raphe n. Vertes et al. 1999
Supralemniscal n. (SuL) (Törk 1990; Jacobs and Azmitia 1992; Vertes and Crane 1997)	B9	Supralemniscal raphe nn. r1SuL r2SuL r3SuL	Caudal portion of the pontis oralis n. (Törk and Hornung 1990; Hornung 2003)
Raphe pontis n. (PnR) (Olszewski and Baxter 1954; Taber et al. 1960; Valverde 1962; Steinbusch 1981; Törk and Hornung 1990; Harding et al. 2004)	B5	Pontine raphe nn. r3PnR r4PnR	Pontine raphe n. (Brown 1943)
			Rostral raphe magnus n. (Taber et al. 1960; Skagerberg and Björklund 1985; Hornung and Fritschy 1988)
Extraraphe cells (Olszewski and Baxter 1954)	B4	Supragenual raphe nn. r5SGeR r6SGeR	Supragenual n. (Paxinos and Watson 2007)
Raphe magnus n. (RMg) (Meessen and Olszewski 1949; Taber et al. 1960; Valverde 1962; Lidov and Molliver 1982; Törk and Hornung 1990; Paxinos and Franklin 2007; Paxinos and Watson 2007)	B3	Raphe magnus nn. r5RMgD r6RMgD r5RMgV r6RMgV	Rostral raphe obscurus n. (Taber et al. 1960)
			Central inferior raphe n. (Marburg 1910)
			Magnocellular nucleus ventralis raphe (Winkler and Potter 1914)
		Parapyramidal raphe nn. (retropontine parts) r5PPy r6PPy	Lateral paragigantocell. (Jacobs and Azmitia 1992)
			Rostral ventrolateral (Törk 1990; Harding et al. 2004)
Raphe obscurus n. (ROb) (Olszewski and Baxter 1954; Taber et al. 1960; Valverde 1962; Steinbusch and Nieuwenhuys 1985; Hornung and Fritschy 1988; Nieuwenhuys et al. 2008)	B2	Raphe obscurus nn.	Posterior raphe group Ramón y Cajal 1909
		r7–r11ROb	Raphe parvus n. (Meessen and Olszewski 1949)
Raphe pallidus n. (RPa) (Olszewski and Baxter 1954; Taber et al. 1960; Valverde 1962; Steinbusch and Nieuwenhuys 1985; Hornung and Fritschy 1988; Nieuwenhuys et al. 2008)	B1	Raphe pallidus nn. r7–r11RPa	Ventral nucleus. Winkler and Potter (1914)
		Parapyramidal raphe nn. (medullary parts) r7–r11PPy	Caudal ventrolateral. (Törk^1990^; Harding et al. 2004)

**Fig. 1:**
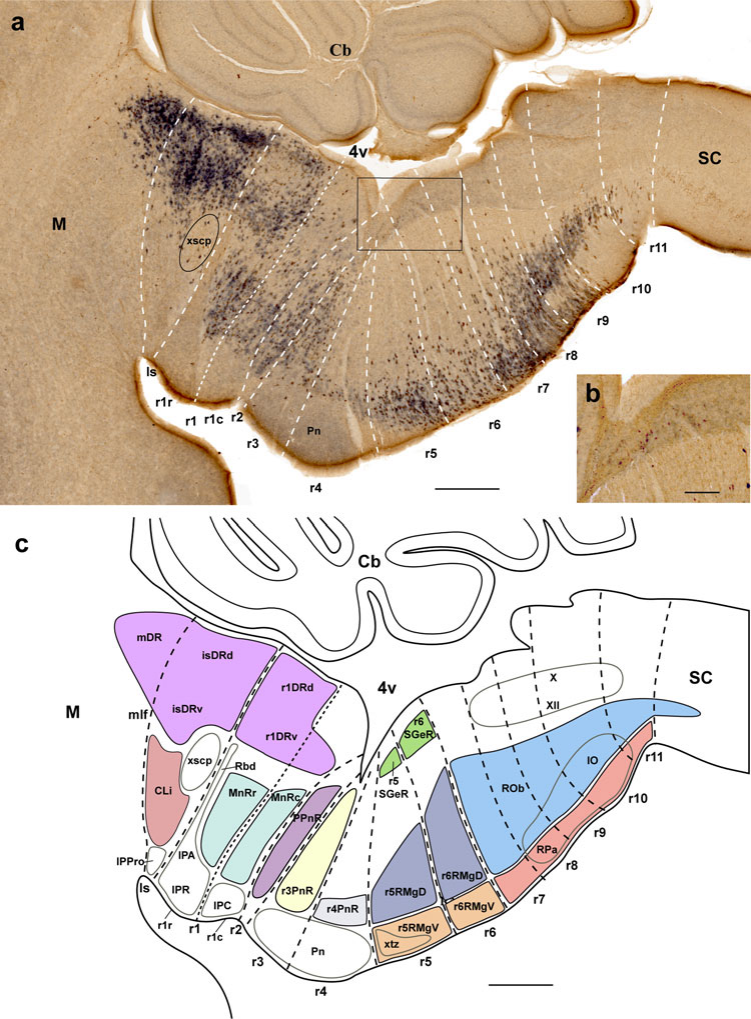
Partial overview of mouse raphe nuclei in a paramedian sagittal section at P10. **a** The staining involves 5-HT-immunoreaction (*brown*) plus *Pet1* in situ hybridization (*blue*). Interrhombomeric boundaries are drawn as *white dashed lines* (*smaller dashes* separate the rostral and caudal halves of r1). **b** Higher magnification of the periventricular area *boxed* in **a**, showing the 5-HT-positive cells of the supragenual raphe cells in r5–r6. **c** Schema according to **a**, interpreting the topological and topographic relations of the illustrated raphe nuclei within the rhombomeric map. A *color-code* was applied to facilitate group distinction. Some characteristic adjacent grisea are indicated as contours for topographic reference. Note that laterally placed raphe nuclei are not shown in this figure. For abbreviations see "List of abbreviations". *Scale bar* 500 μm in **a** and **c**, and 150 μm in **b**

Our present aim is to advance a complete rhombomeric classification of raphe nuclei, expecting that this may help causal neuromeric analysis of shared and differential aspects of their molecular specification and differentiation. Our approach suggests a modified terminology that contemplates such developmental ascription (Table 1; Fig. 1b). In our analysis, apart of attending to literature data on genoarchitecture, fate mapping (Marín and Puelles 1995; Cambronero and Puelles 2000) and rhombomere-related lineage mapping (Jensen et al. 2008), we essentially followed the rhombomere schema of the Allen Developing Mouse Brain Atlas (http://developingmouse.brain-map.org/), whose reference atlases indicating rhombomeric units at different stages oriented our interpretation (note these reference atlases were elaborated by LP; see similar use by Watson and Paxinos 2010). Such mapping is relatively straightforward and reproducible, due to the abundance of known neuromeric landmarks. A further point of interest was to check whether the molecular profile of developing raphe populations is uniform along the diverse hindbrain neuromeric units, or shows some regional differences, irrespective of the development of a common neurotransmitter phenotype. To this end, we mapped comparatively in sagittal sections at critical developmental stages diverse gene markers previously associated to specification of the serotonergic neuronal phenotype (*En1*, *En2*, *Gata2*, *Gata3*, *Lmx1b*, *Pet1*, *Slc6a4* and *Tph2*).

At early stages, some rhombomeric limits are identifiable as constrictions of the neural tube wall, though these flatten out as development advances and the neural wall thickens. However, there are also so-called crypto-rhombomeres in the medulla oblongata, whose interneuromeric limits are not morphologically distinguishable; these units were first found in the chick via experimental fate-mapping studies (Cambronero and Puelles 2000; in that report they were named ‘pseudo-rhombomeres’, but the more apt name ‘crypto-rhombomeres’ was thereafter suggested by R. Nieuwenhuys—personal communication to LP). The existence of crypto-rhombomeres was subsequently corroborated by the observation of corresponding molecular limits, namely step-like arrangement of the rostral borders of expression of *Hox* genes of the 4–8 paralogous groups, analogously to the patterns of paralogous *Hox* gene groups 1–3 across the overt rhombomeres (Marín et al. 2008). There are reasons to assume that the mouse medulla has the same hidden partitions (Holstege et al. 2008; Watson et al. 2010; Allen Developing Mouse Brain Atlas; Puelles 2012, in press). To corroborate our identification of interrhombomeric boundaries, we compared our 5-HT-immunoreacted or hybridized sagittal sections with equivalent sections with mapped homeobox gene expression patterns found in the Allen Atlas database, thus correlating our data with the relevant molecular boundary landmarks (data not shown). Such expression patterns were more useful at early embryonic stages (E10.5 to E14.5), since at later stages (E16.5 to P10) many marker genes gradually downregulate their expression. However, the late developmental period is precisely when anatomical landmarks with known rhombomeric location become more distinct (nerve roots, characteristic nuclei with known neuromeric position, various decussations).

In general, the major groupings or aggregates of raphe neurons were sufficiently discrete that their neuromeric position could be resolved with reasonable reproducibility. Comparison with available literature on the connections of the raphe nuclei suggests that differential raphe projections are indeed arranged segmentally and may thus have a fundament in the differential molecular identities of the rhombomeric raphe units. Moreover, our analysis of the developmental emergence of characteristic raphe molecular typology revealed some interesting regional differences.

It has been previously reported that serotonin deficiency is a relatively common finding in neuropaediatric patients with different congenital disorders, including sudden infant death syndrome, fetal alcohol syndrome and autism (Jensen et al. 2008; De Grandis et al. 2010). However, etiological diagnosis is not achieved in most cases. This suggests that investigations of genes and histogenetic mechanisms involved in the development and maturation of functional raphe nuclei may provide in the long run new insights on the etiology of impaired serotonin transmission in the central nervous system.

## Materials and methods

### Animals

All mice were treated according to the stipulations and laws of the European Union (86/609/EEC) and the Spanish Government (Royal Decree 223/1998) on the care and handling of research animals. The strain used was Swiss Albino. The day of the vaginal post-coital plug formation was regarded as embryonic day 0.5 (E0.5). All procedures were performed according to protocols approved by the University of Murcia Committee for Animal Experimental Ethics. Embryos from E12.5 to E18.5 and postnatal mice from P0 to P10 were used (six embryos or mice per stage).

### Tissue preparation

Embryos at E12.5 and E14.5 (stage corroborated according to Theiler 1989), were killed and fixed by immersion in phosphate-buffered 4 % paraformaldehyde (0.1 M PB; pH7.4) at 4 °C for 24 h. E18.5 embryos and postnatal animals were anesthetized on ice and perfused transcardially with PB and the fixative solution. The brains were then dissected out and postfixed for 24 h at 4 °C.

For in situ hybridization (ISH), the brains were embedded in 4 % agarose in PBS (phosphate-buffered saline solution) and sectioned 80-μm thick in the sagittal or coronal planes using a vibratome (Leica Microsystems, Nussloch, Germany).

### Reverse-transcription polymerase chain reaction (RT-PCR)

In order to obtain the cDNA of mouse *Gata2* and *Gata3* genes, we extracted RNA with Trizol reagent (Invitrogen, La Jolla, CA, USA) from freshly dissected mouse brains at E12.5 and E14.5 stages. The RNA was treated with DNase I (Invitrogen) for 15 min at room temperature, followed by enzyme inactivation at 65 °C. The cDNA was obtained by reverse transcription from RNA with Superscript II reverse transcriptase and random hexamer primers (SuperScript First-Strand Synthesis System for RT-PCR; Invitrogen).

The resulting first-strand cDNA (0.5 μL of the reverse transcription reaction) was used as a template for PCR, performed with Taq polymerase (M8305, Promega, Madison, WI) and specific primers for *Gata2* (forward primer: 5′-cttcctccagtctctcttttgg-3′, reverse primer: 5′-tacaccagctttggcctctg-3′) and *Gata3* (forward primer: 5′-ctgcaaaccattaaacga-3′, reverse primer: 5′-acgtctccagcttcatgctatc) mRNAs. PCR conditions were as follows: 5 min at 94 °C, then 35 cycles (30 s at 94 °C, plus 1 min at *T*_m_ temperature, 58 °C, and 1 min at 72 °C), followed by 10 min at 72 °C. The PCR products were cloned into pGEM-T Easy Vector (Promega) and sequenced (SAI, University of Murcia).

### In situ hybridization

The *En1* (NM010133.2, positions 147-2032), *En2* (NM 010134.3, positions 1316-2098), *Lmx1b* (NM 010725.1, positions 1-898), *Otx2* (NM 144841.2, positions 592-1165) and *Pet1* (NM 153111.1, positions 897-1396) riboprobes were synthesized from plasmids kindly provided by K. Schughart (*En1*), J. Rossant (*En2*), R. Johnson (*Lmx1b*), A. Simeone (*Otx2*) and W. Wurst (*Pet1*). The riboprobes *Tph2* and *Slc6a4* were supplied by ImaGenes (Berlin, Germany) from their Mouse EST collection (*Tph2,* clone RZPDp981F09257D, NM 173391.1, positions 47-649; *Slc6a4*, clone RZPDp981H09201D, NM 010484.1, positions 59-389). The *Gata2* (NM 008090, positions 1680-2441) and *Gata3* (NM 008091.2, positions 68-785) riboprobes were synthesized from cDNA cloned at our laboratory. All cDNA used in this work was sequenced (SAI, University of Murcia) and specificity was checked using the BLAST tool (NCBI).

The hybridizations on floating vibratome-sections were done according to the protocol of Shimamura et al. (1994). As general in situ hybridization (ISH) controls, sense and antisense probes were applied to adjacent representative sections (the signal was present only with antisense probe), and some sections were processed without either sense or antisense probes, to check for possible background due to the other reactives used in the standard ISH procedure. To detect the hybridized product, the sections were incubated overnight with alkaline phosphatase-conjugated antidigoxigenin Fab fragments (1:3,500, Roche Diagnostics, Manheim, Germany), and nitroblue tetrazolium/bromochloroindolyl phosphate (NBT/BCIP) was used as chromogenic substrate for the final alkaline phosphatase reaction (Boehringer, Mannheim, Germany).

### Immunohistochemistry

All immunoreacted sections were processed following the same free-floating protocol, including those processed after ISH. Sections were washed in PBS and then treated with 0.1 % hydrogen peroxide in PBS for 1 h in the dark to inactivate endogenous peroxidase activity. After several rinses in PBT (PBS with 0.2 % Triton X-100), sections were blocked with 0.5 % goat serum, 0.2 % bovine serum albumin (BSA) and 0.2 % Triton X-100 (Sigma, St. Louis, MO, USA) in PBS for 4 h, and then, incubated overnight at 4 °C with polyclonal rabbit anti-5-HT antibody (1:1,000; ImmunoStar, Hudson, USA; cat. no 20080), prepared in the same blocking solution. This primary reaction was developed with biotinylated goat anti-rabbit secondary antibody (1:200, 2 h of incubation; Vector Laboratories, Burlingame, CA, USA), and then with streptavidin/horseradish peroxidase (HRP) complex (1:200, 2 h of incubation; Vectastain-ABC kit; Vector Laboratories, Burlingame, CA, USA). All antibodies were diluted in the same blocking solution as the primary antibody. The histochemical detection of the peroxidase activity was carried out using 0.03 % diaminobenzidine (DAB) and 0.005 % H_2_O_2_. After immunoreaction, the sections were mounted, dehydrated and then coverslipped with Eukitt (Fluka, Buchs, Switzerland).

### Antisera characterization

The commercial rabbit anti-5-HT polyclonal antibody employed in this report was raised against serotonin derived from rat brain, coupled to bovine serum albumin (BSA) with paraformaldehyde (Immunostar, Hudson, WI, USA; catalog number 20080; manufacturer’s technical information). This antibody was shown to recognize specifically serotonin molecules (5-hydroxytryptamine) in mouse brainstem (Fortune and Lurie 2009). The 5-HT immunoreactions obtained in our mouse brainstem tissue reveal a virtually identical staining pattern as the *Pet1* riboprobe, which is a specific marker of the serotonergic neuronal phenotype (Hendricks et al. 1999). Moreover, pre-adsorption of the diluted antibody with 25 μg/mL of serotonin/BSA complex eliminated completely the reaction, whereas pretreatment with BSA did not affect the immunostaining.

### Imaging

Digital microphotographs were obtained with a Zeiss Axiocam camera (Carl Zeiss, Oberkochen, Germany) or with a ScanScope digital slide scanner (Aperio, Vista, CA, USA) and the images were corrected for contrast and brightness using Photoshop CS3 (Adobe Systems, San Jose, CA, USA). All plates were produced and labeled in Adobe Illustrator CS3 software (Adobe Systems, San Jose, CA, USA).

## Results

### Generalities

**Fig. 2:**
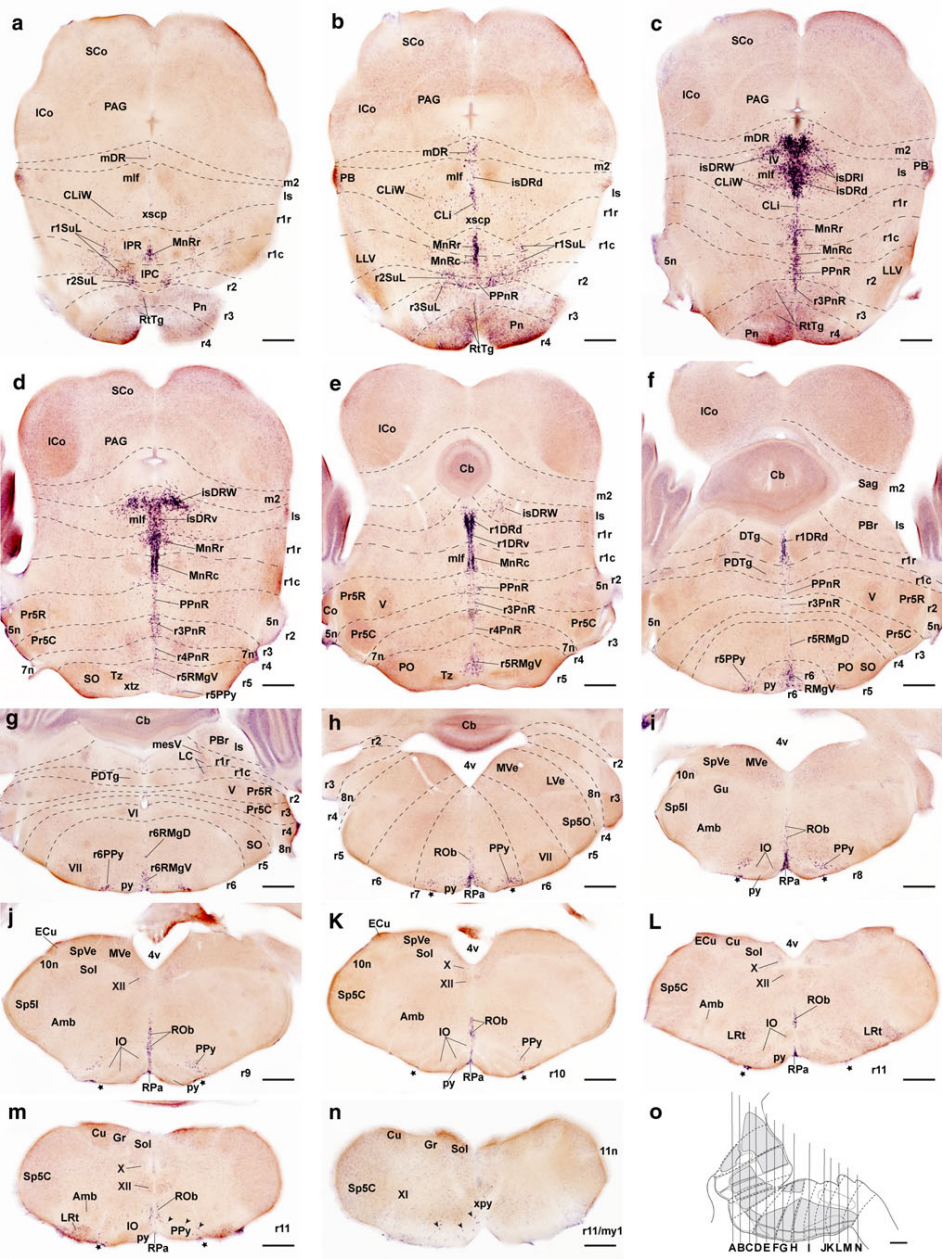
*Pet1* expression detected by in situ *hybridization* across the midbrain–hindbrain continuum, shown in a series of 14 non-consecutive standard ‘cross sections’ at P10 (these are first horizontal and then transversal to the rhombomeres; see schema of section levels and planes in **o**, based on Fig. 1c). The rhombomeric boundaries are traced as *thin dashed lines*. Selected other structures are identified for topographic reference. **a–c** Levels through rostral midbrain and ventral parts of prepontine/pontine hindbrain (sections are here distinctly horizontal). **d–f** Levels through caudal midbrain, dorsal parts of prepontine/pontine hindbrain and rostral cerebellum (sections are still largely horizontal). **g–n** Levels through retropontine and medullary hindbrain (transversal sections). **o** Schema representing the different levels of section shown in **a–n**. *Black stars* in **h–m** mark subpial *pet1*-positive cells found superficial to the pyramidal tract (py). *Arrowheads* in **m** and **n** point out *pet1*-positive cells associated to the pyramidal decussation (xpy). For abbreviations see "List of abbreviations". *scale bar* 350 μm in **a–n**, and 150 μm in **o**

**Fig. 3:**
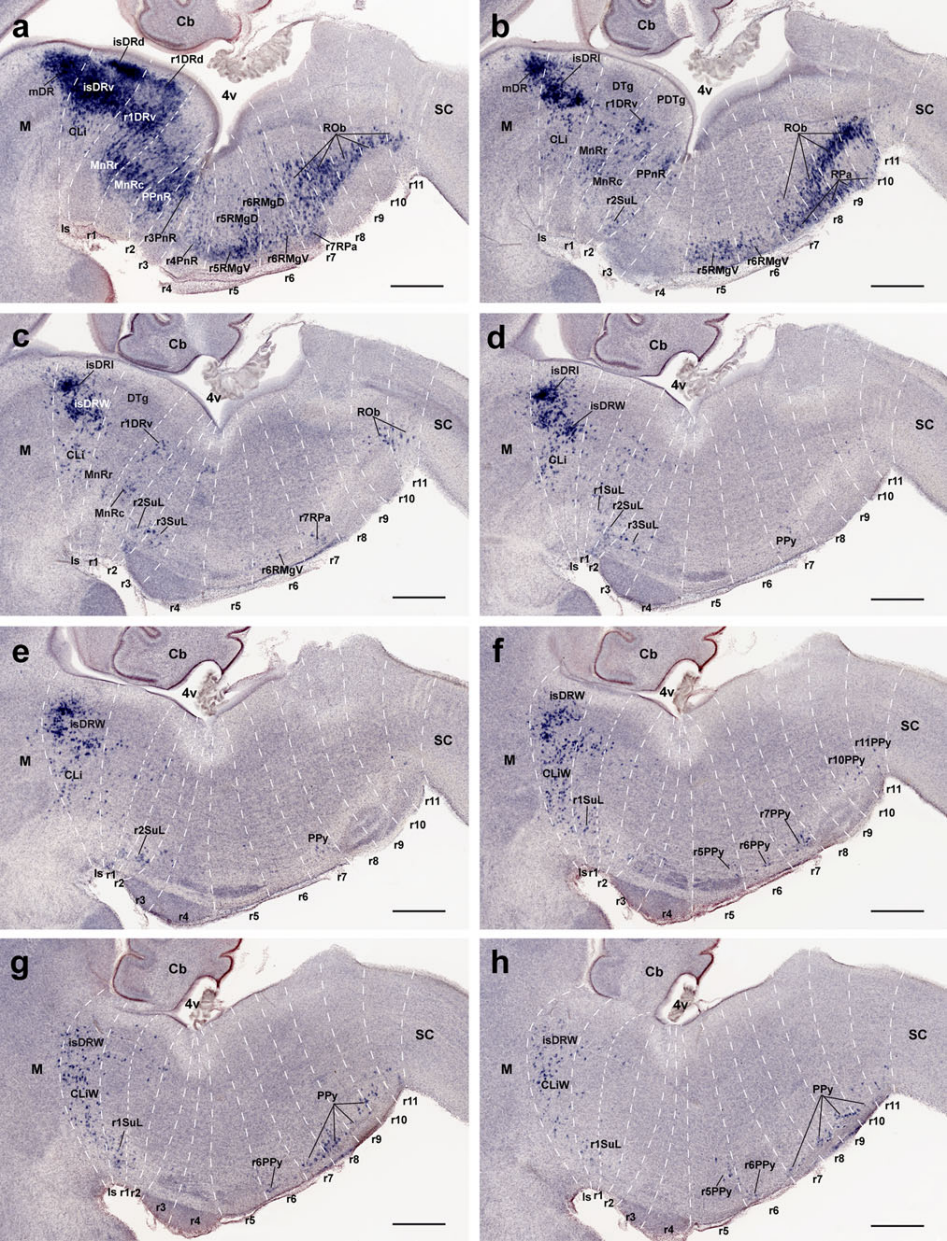
*Pet1* detected by in situ *hybridization* in a series of consecutive sagittal sections proceeding from medial to lateral at P0. The rhombomeric boundaries are traced as *white dashed lines* (*smaller dashes* separate the rostral and caudal halves of r1). **a**, **b** Paramedian levels; compare **a** with the P10 distribution shown in Fig. 1a. **c–h** More lateral levels, showing the migrated wing and ventrolateral raphe formations. For abbreviations see "List of abbreviations". *Scale bar* 500 μm

We first addressed serotonergic cell distribution at P0 and P10, when the morphology of raphe nuclei is essentially definitive (Figs. 1, 2, 3; Watson and Paxinos 2010). The traditional mammalian raphe nuclei (Taber et al. 1960, and other authors; see Table 1), or B1 to B9 groups of Dahlström and Fuxe (1964), were assigned tentatively to individual rhombomere-derived territories. A small rostral part of the dorsal raphe nucleus maps within the caudal midbrain (Fig. 1); see comments on this below.

According to the schema of rhombomeres employed in the Allen Developing Mouse Brain Atlas, which is based on the experimental fate-mapping or *Hox*-gene-mapping works of Marín and Puelles (1995), Cambronero and Puelles (2000), Marín et al. (2008), and Holstege et al. (2008), we contemplate 12 rhombomeric units, ranging from the isthmus (r0) to r11, next to the rhombo-spinal junction. Note there are authors that alternatively encompass our r8–r11 units within a large ‘r8’ rhombomere, and also lump our Is-r1 units into an extra-large ‘r1’ domain, due to variant criteria about how to define a rhombomere (Lumsden and Krumlauf 1996; compare Puelles and Rubenstein 2003; Puelles 2009a, b); this controversy is irrelevant for our present purpose, since readers can lump our conclusions according to their own rhombomeric schema. We agree with Vaage (1969, 1973) in distinguishing rostral and caudal parts of r1 (r1r, r1c; Fig. 1). Both Puelles et al. (2007) and the Allen Developing Mouse Brain Atlas propose prepontine (Is, r1–r2), pontine (r3–r4), pontomedullary or retropontine (r5–r6) and medullary (r7–r11) hindbrain subregions. Raphe nuclei have been subdivided classically into rostral and caudal clusters (Lidov and Molliver 1982; Aitken and Törk 1988), which correspond to blocks developing within Is-r4 and r5–r11, respectively. It is often stated in the literature that r4 lacks altogether a serotonergic raphe population (e.g., Pattyn et al. 2003). However, we do observe a small population at this locus (e.g., Fig. 1).

In Fig. 1, we mapped serotonin immunoreaction jointly with ISH for the gene *Pet1*, a serotonergic marker, at P10. Visibly, postnatal serotonergic neurons are not grouped uniformly along the rostro-caudal series of rhombomeres. In some rhombomeres (e.g., r4) there is only a sparse raphe population, whereas in others we find several separate dense aggregates (e.g., in r1). These are described in detail in the next section. The serotonergic nuclei mainly occupy the paramedian basal region of the rhombomere they belong to (only occasionally penetrating secondarily the median raphe region proper, which is a cell-poor, floorplate-derived, astroglial palisade). Individual raphe populations differ in their radial position (stratification) within the paramedian basal plate, occupying positions at either the periventricular, intermediate or superficial strata. In addition, there is also some mediolateral dispersion. Some serotonergic elements appear displaced laterally from the raphe neighborhood, at periventricular, intermediate or superficial positions, but nevertheless remain always within the basal plate.

### Rhombomeric pattern of mouse raphe nuclei at P0 and P10

#### The rostral cluster

This cluster is characterized by a stereotyped distribution of its elements in the periventricular, intermediate and superficial strata, largely within the four rostralmost rhombomeres (Is, r1–r3), to which must be added the mentioned minor population in r4 and the small subpopulation found within the periaqueductal gray of the caudal midbrain (Figs. 1, 2a–f, 3). We will deal separately with the latter.

##### Isthmic raphe nuclei

The present identification of these elements rests upon the capacity to identify the isthmic territory of the brainstem as distinct from the midbrain (see Puelles et al. 2012b) and rhombomere 1 (note that several authors assume that the isthmus is merely a vaguely defined rostral part of r1; see “Discussion”). We used *Otx2* expression selectively present in the midbrain to identify the rostral boundary of the Is (Simeone et al. 1992, 1994). The caudal isthmic limit was estimated by the selective presence of numerous *Pax7,* *Pax3* and *Otp* expressing cells in the r1 mantle; these do not extend into the Is mantle (Allen Developing Mouse Brain Atlas, http://developingmouse.brain-map.org/). Additional isthmic landmarks are provided by the ventricular isthmic fossa at the midline floor area, plus the paramedian trochlear motor nucleus, which jointly allowed us to delimit the Is tegmentum from that of the ventral midbrain (Palmgren 1921; Vaage 1969, 1973; Kuhlenbeck 1973). Moreover, the decussation of the superior cerebellar peduncle (xscp) was estimated to lie just rostral to the Is/r1 boundary as defined by the mentioned gene markers. The isthmic serotonergic populations are represented mainly by what we call the *isthmic dorsal raphe nucleus* (isDR), which is the largest part of the conventional dorsal raphe nucleus (commonly wrongly held to lie in the midbrain; compare Fig. 1); this is composed of dorsal, ventral, lateral and wing subdivisions (isDRd, isDRv, isDRl, isDRW). The isDR is complemented by a much sparser serotonergic population found at the caudal linear nucleus (CLi), including laterally dispersed cells, or wing portion of the CLi (CLiW).

The isDR is easily recognizable in paramedian sagittal sections, because it shows a considerable density of its 5-HT-positive cells, contrasting with its caudal neighbor, the less populated *r1 dorsal raphe nucleus* (r1DR) (Figs. 1a–c, 2b–f, 3a). The isDRd occupies the ventral midline of the isthmic periaqueductal stratum, while the isDRv lies ventral to the former at the corresponding deep intermediate stratum, just medial to the trochlear motor nucleus; the isDRl caps bilaterally the trochlear nucleus and the medial longitudinal fascicle (IV, mlf; Figs. 1, 2b–d, 3a–d). Similar neurons appear dispersed even more laterally, in what may be classified as wings of the isDR (isDRW); these cells lie either inside or outside the periaqueductal gray (ventrolateral DR of Paxinos and Franklin 2007). Comparison of isDR with the isthmo-mesencephalic boundary (passing in sagittal sections between the oculomotor and trochlear nuclei; Palmgren 1921; Vaage 1973; Joyner et al. 2000; Zervas et al. 2004; Puelles et al. 2012b) strongly suggests that a rostral subgroup of 5-HT-positive cells of the DR complex actually lies within the caudal midbrain (Figs. 1a, 3a, b). Puelles et al. (2012b) have recently proposed that this midbrain DR formation lies specifically at the periaqueductal midline within the mesomere 2, that is, at the caudalmost, preisthmic developmental subregion of the midbrain, a locus also rich in dopaminergic neurons. We will examine below the issue whether these cells arise in the midbrain, or result from a migration originated in the isthmus, as was already suggested by Zervas et al. (2004). In any case, we named this rostral pole of the DR complex the *midbrain dorsal raphe nucleus* (mDR), according to its adult topography (note the literature often takes the entire DR complex as being mesencephalic; see “Discussion”).

The CLi is populated by dispersed serotonin-immunoreactive neurons distributed at intermediate radial levels of the isthmic paramedian tegmentum (they do not extend into the superficially placed isthmic part of the interpeduncular nucleus; Fig. 1). This paramedian isthmic tegmental area is intercalated rostrocaudally between the dorsal and ventral tegmental decussations of the midbrain and the decussation of the superior cerebellar peduncle found within caudal Is. The CLi cells are less abundant and compact than those of the isDR, and do not invade the midbrain (Figs. 1a, 2b, c, 3a–e). Laterally to the CLi nucleus, a serotonergic wing-like population appears, with similar cell density than the CLi. We named this serotonergic group *the caudal linear wing nucleus* (CLiW; Fig. 2b, c).

##### R1 raphe nuclei

**Fig. 4:**
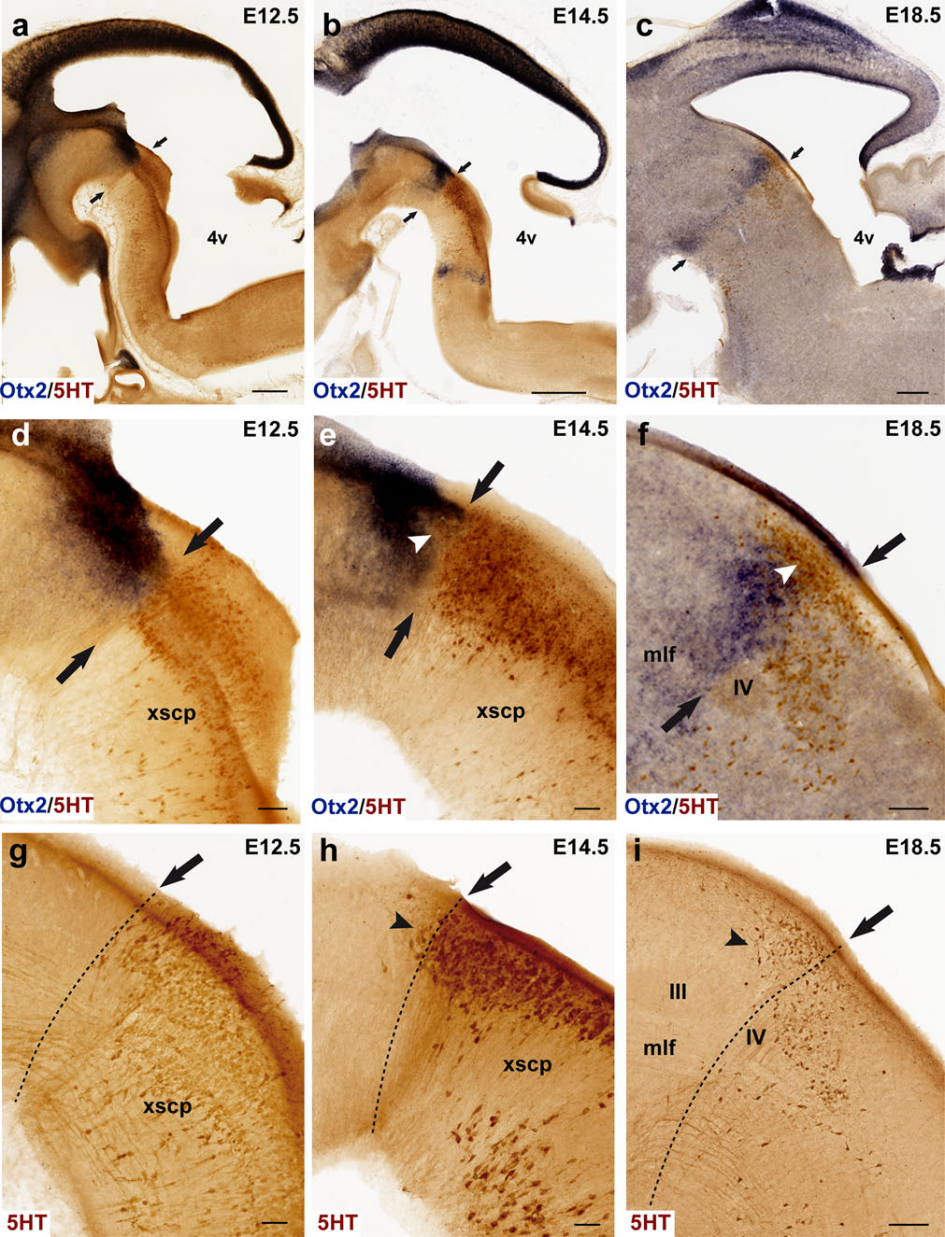
Late appearance of the mesencephalic part of the dorsal raphe nucleus. The issue whether any raphe neurons arise in the midbrain is approached here by comparing 5-HT-immunoreaction with *Otx2* in situ hybridization on the same sections, at different stages (*Otx2* is known to mark sharply the caudal boundary of the midbrain). **a–c** Low-magnification images of 5-HT (*brown*) and *Otx2* (*blue*) signals at the hindbrain–midbrain boundary in paramedian sagittal sections at three different developmental stages. *Arrows* indicate the midbrain/hindbrain boundary, as marked by *Otx2* expression (consistently with parallel fate-mapping data). **d–f** Higher magnification of the sections in **a–c**, respectively, showing details of the changing topographic relationship of serotonergic neurons with the midbrain boundary. No midbrain 5-HT cells are observed at E12.5 (**d**), but a few become apparent at E14.5 (**e**); these occupy a small triangular area (marked by *white arrowhead*) in front of the boundary (*arrows*), where *Otx2* expression seems to be partially downregulated. At E18.5 (**f**) a discrete group of 5-HT-positive cells appears in front of the boundary (*arrows*; note also the isthmic landmark provided by the trochlear nucleus, *IV*); these serotonergic elements are located mostly in a periventricular stratum that is *Otx2*-negative (*white arrowhead*), though the ventrally adjacent *Otx2*-positive periaqueductal gray also shows some dispersed serotonergic neurons. This cell group is interpreted by us as mDR, and lies only in the caudal preisthmic midbrain (mesomere 2; compare **c**). **g–i** Sagittal sections equivalent to those shown in **d**–**f** (same stages, respectively), but illustrate only 5-HT immunoreacted cells (corresponding low-magnification images are shown in Fig. 5a–c). The midbrain–hindbrain boundary is marked by a *dashed line*, and *black arrowheads* indicate the emergent mDR neurons. *4v* fourth ventricle, *III* oculomotor nucleus, *IV* trochlear nucleus, *mlf* medial longitudinal fasciculus, *xscp* decussation of the superior cerebellar peduncle. *Scale bar* 200 μm in **a**, 500 μm in **b**, 400 μm in **c**, 50 μm in **d–e** and **g–h**, 100 μm and in **f** and **i**

The periventricular r1 domain contains what we call the caudal or r1 portion of the dorsal raphe nucleus (r1DR), as well as the median raphe group (MnR) and a large superficial ventrolateral aggregate (r1SuL). The MnR (a.k.a. central superior nucleus; Table 1) extends as defined conventionally also into r2, but for clarity we propose recognizing two distinct nuclei here, reserving the MnR name for r1 (see below as regards r2). The supralemniscal r1 raphe nucleus (r1SuL) corresponds to the larger rostral part of a ventrolaterally displaced cell population that extends likewise into r2 and r3, at least (Table 1). To pinpoint the rostro-caudal limit of r1 and, therefore, of these nuclei, we used as before the selective widespread tegmental distribution of *Pax7* and *Pax3* positive cells in the r1 mantle layer, as well as the selective expression of *Otx2* in a basal paramedian population restricted to the caudal half of r1 (unlabeled radial patch in Fig. 4b; Lorente-Cánovas et al. 2012; Puelles, unpublished observations; Allen Developing Mouse Brain Atlas, http://developingmouse.brain-map.org/). Remarkably, the *Otx2* pattern corroborates molecularly distinct rostral and caudal halves of r1 (a point previously deduced from differential histogenetic patterns by Vaage 1973; this author noted that r1 is about double as large as a normal rhombomere); we identify here these parts as r1r and r1c (Fig. 1). Moreover, the early expression of *Hoxa2* characteristically ends rostrally at the r1–r2 boundary (Irving and Mason 2000; Moens and Prince 2002; Aroca and Puelles 2005; Oury et al. 2006; Lorente-Cánovas et al. 2012; Puelles, unpublished observations).

The r1DR portion of the dorsal raphe nucleus is on the whole less populated than the isthmic portion, and is found largely in paramedian periventricular position within the rostral and caudal parts of r1, though predominantly in the rostral part (r1DR; Figs. 1, 2e, f, 3a). In contrast to the isDR, the r1DR shows only a sparse laterally placed wing portion (r1DRW), but dorsal (periaqueductal) and ventral (intermediate) subnuclei can be distinguished, as in the isthmus (r1DRd; r1DRv; Figs. 1, 2e, f, 3a); the r1DRv is the only component that extends into r1c (Fig. 1b). Its periaqueductal or dorsal cell mass is intercalated at the midline between the bilateral dorsal tegmental nuclei, whereas the ventral portion separates similarly the ventral tegmental in r1c nuclei (DTg, VTg; Figs. 1, 2e, f, 3a); the r1DRv relates to the posterodorsal tegmental nucleus (PDTg; Fig. 15). There are very few midline serotonergic cells at the caudal half of r1, next to the posterodorsal tegmental nucleus (Figs. 1, 3a, 15). It does not seem meaningful to identify these sparse caudal r1 neurons as a straightforward raphe nucleus population, associated or not to the DR.

In contrast, the median raphe nucleus (MnR), presently redefined by us so that the r2 analog is excluded from this concept, occupies both halves of r1; there is no obvious separation between the r1r and r1c moieties at postnatal stages. This cell group lies across the median glial raphe at intermediate levels of the r1 tegmentum, deep to the r1 parts of the interpeduncular nucleus (MnR, IPR, IPC; Fig. 1). Its neurons appear sharply separated from the periaqueductal r1DR formation (Figs. 1, 2a–f, 3a–d), though sparser serotonergic cells can be found occasionally in-between. The r1r part of MnR (MnRr) respects rostrally the paramedian space occupied by the apical interpeduncular and rhabdoid nuclei, and similarly respects superficially (ventrally) the space occupied by the rostral interpeduncular nucleus (IPA, Rbd, IPR; Fig. 1). The MnRr is slightly larger than its r1c counterpart (MnRc), and apparently contains more 5-HT-immunoreactive cells (Figs. 1, 2c–f, 3a). The MnRc lies medial to the *Otx2*-positive tegmental population in the paramedian basal plate of caudal r1 (data not shown; see Allen Atlas; Lorente-Cánovas et al. 2012), and is separated from the pial surface by the caudal interpeduncular nucleus (IPC; Figs. 1a, 2a, 3a).

Apart of MnR, there is also a sizeable population of r1 serotonergic cells that appear displaced laterally from the median raphe neighborhood. Such elements are rather dispersed at intermediate radial levels, where they appear typically just outside of the paramedian locus identified classically as ‘paramedian raphe nucleus’ (itself devoid of serotonergic neurons). Similar lateral cells tend to form a larger aggregate more superficially (ventrally), abutting the rostral part of the decussation of the trigeminal lemniscus (therefore the ‘supralemniscal’ descriptor applied by some authors; Table 1), lateral to the interpeduncular nucleus. Given that similar lateral cells appear as well in r2 and r3, we identify it as the *supralemniscal r1 raphe nucleus* (r1SuL; Figs. 1, 2a–b, 3d). Similar supralemniscal raphe populations are distinguished in r2 and r3 (see below).

##### R2 raphe nuclei

The r2 basal plate is delimited rostrally by the *Pax7*/*Pax3*/*Otx2*-expressing cells of the caudal interpeduncular subnucleus and overlying basal tegmentum within r1c (see above), and caudally by the pontine nuclei, the related pontine decussation (xpn) and the reticulotegmental nucleus in r3–r4 (Puelles et al. 2007; Fig. 1). In postnatal mice, the r2 floorplate is much compressed rostrocaudally between the IPC (r1c) and the basilar pons in r3–r4; it typically contains the major part of the decussation of the trigeminal lemniscus. We compared the location of these landmarks with the expression pattern of *Hoxa2* and *Hoxb2* genes from the Allen Developing Mouse Brain Atlas (http://developingmouse.brain-map.org/). The expression domains of *Hoxa2* and *Hoxb2*, respectively, stop rostrally at the r1/r2 and r2/r3 boundaries. *Hoxa2* ISH signal abuts rostrally the *Otx2*-positive r1 domain.

We commented above that the caudal portion of the classic MnR nucleus lies across the r2 raphe. However, it seems convenient to separate this cell population from the MnR proper (in r1), as is suggested by some differential markers (see below). We therefore called the distinct r2 population of raphe serotonergic cells the *prepontine raphe nucleus* (PPnR), on account of its topography relative to the pontine rhombomeres r3–r4. The major part of PPnR lies across the midline raphe at intermediate radial levels of r2, starting ventrally just dorsal to the trigeminal lemniscal decussation (PPnR; Figs. 1, 2b–d, 3a–c).

In addition, there are also ventrolaterally displaced superficial serotonergic cells within r2, which we have named the *supralemniscal r2* *raphe nucleus* (r2SuL; Fig. 2); this cell group is smaller than r1SuL. These neurons are aligned longitudinally with the r1 and r3 counterparts (Figs. 2a–b, 3d–e).

##### R3 and r4 raphe nuclei

For these neuromeres we used as medial landmarks the pontine and reticulotegmental nuclei (Pn, RtTg), which invade these two developmental units from E15.5 onwards, after culminating their respective tangential migrations from the rhombic lip. The trigeminal sensory and motor nerve roots emerge laterally at the caudal end of r2, aiding the delimitation from r3 (Allen Developing Mouse Brain Atlas, http://developingmouse.brain-map.org/) and the rostral end of the *Hoxb2* expression domain likewise delimits r2 from r3 (*Egr2*—known before as *Krox20*—labels selectively r3 and r5, being likewise useful for the present purpose). Moreover, the roots of the facial and vestibulocochlear nerves traverse laterally r4 (contained wholly within its rostral and caudal limits), and the intraneural course of the facial motor fibers within r4 (rostral to the genu) also allows a rough estimation of the r3–r4 and r4–r5 boundaries. Both r3 and r4 are considerably compressed rostrocaudally near the fourth ventricle, but expand anteroposteriorly at the subpial pontine basilar complex (Fig. 1).

**Fig. 5:**
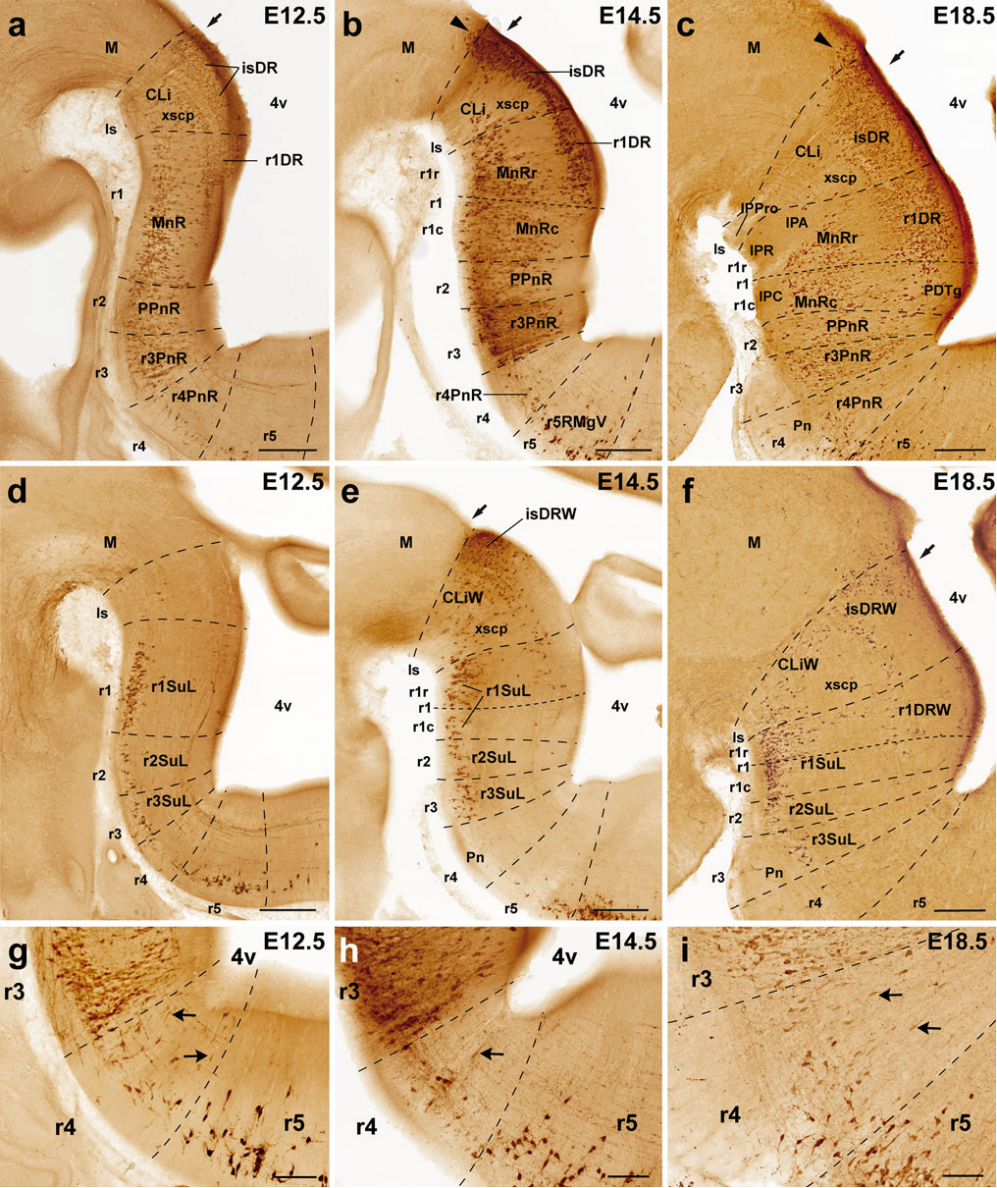
Segmental mapping of the rostral raphe cluster during embryonic development. 5-HT immunoreactive neurons observed in sagittal sections of mouse brains at E12.5 (**a**, **d**), E14.5 (**b**, **e**), and E18.5 (**c**, **f**), with superposed tracing of postulated interrhombomeric boundaries (*dashed lines*), and our tentative identification of the nuclear primordia (Table 1). Each set of three images read from *left* to *right* (e.g., **a**–**c**) represents a temporal sequence at a given section plane. *Arrows* mark the midbrain–hindbrain boundary. **a–f** Rostral cluster at paramedian section level. **d–f** Rostral cluster at a more lateral level. **g–i** Details at higher magnification of the paramedian pontine region of Fig. 5a–c, respectively. Note some 5-HT immunopositive cells are always present in r4, mainly in its superficial stratum, though cells with weaker immunoreaction are also observed in the intermediate stratum (*arrows* in **g**–**i**). For abbreviations see "List of abbreviations". *Scale bar* 250 μm in **a–f**, 100 μm in **g–i**

We found that the r3 raphe sector clearly contains more serotonergic cells than r4, though possibly less than r2 (Figs. 1, 2c–e, 3a). The r3 paramedian elements represent a distinct, radially elongated *pontine r3 raphe nucleus* (r3PnR) that extends through intermediate radial levels, immediately caudal to the PPnR in r2 (r3PnR; Figs. 1, 2c–e, 3a). More superficial, laterally displaced serotonergic neurons constitute separately the r3SuL cell group, which appears dorsal to the r3 basilar pons, partly associated to the reticulotegmental nucleus (r3SuL; Figs. 2b, 3c–d); note that at embryonic stages these cells lie subpially (Fig. 5a, b, d, e), but apparently result covered by the pontine and reticulotegmental migrations. We found no counterpart of r3SuL within r4.

Serotonin cells are much reduced in number in r4, but some cells are nevertheless clearly present. Most of these cells are characterized postnatally by low 5-HT-immunoreaction (or low *Pet1* signal), but we also observed a few highly 5-HT-immunoreactive (and *Pet1* expressing) scattered neurons (Fig. 1a). We identified the small paramedian serotonergic aggregate lying at a ventral intermediate radial level just dorsal to the pontine nuclei as the *pontine r4 raphe nucleus* (r4PnR; Figs. 1, 2d, e, 3a). This validates the expression ‘pontine raphe nuclei’, restricted to the paramedian raphe components of r3 and r4. Note, however, that this expression has been wrongly used in the literature, applying it to raphe elements lying more rostrally (even up to the Is), due to the misconception that the entire rostral hindbrain is ‘pontine’ (Table 1 and “Discussion”).

#### The caudal cluster

Traditionally the caudal cluster is held to be formed by the raphe magnus (RMg), the raphe obscurus (ROb) and the raphe pallidus (RPa) nuclei (e.g., Taber et al., 1960), plus a set of lateralized superficial parapyramidal neurons. These formations represent plurisegmental complexes, once their respective topography relative to the r5–r11 developmental units is taken into account. The most rostral of these populations, the RMg, appears to be distributed across rhombomeres r5 and r6 (these are the ‘retropontine’ rhombomeres according to Puelles et al. 2007, or the ‘pontomedullary’ rhombomeric units of the Allen Developing Mouse Brain Atlas), whereas the more caudal ROb and RPa complexes are both distributed rostrocaudally all along the caudal medulla oblongata, which is held to be subdivided into five cryptorhombomeres (r7 to r11) (Cambronero and Puelles 2000; Puelles et al. 2007; Marín et al. 2008). The parapyramidal neurons extend throughout the r5–r11 continuum.

##### R5 and r6 raphe nuclei

The superficial trapezoid body decussation (xtz) and associated auditory grisea (particularly the nucleus of the trapezoid body), which are restricted to r5, jointly with the facial motor nucleus, that characteristically is located in r6 (after its early migration), allowed us to pinpoint the r5/r6 boundary (Figs. 1, 2d–e; Wolfer et al. 1994; Puelles et al. 2007; Marín et al. 2008). In addition, this limit coincides with the rostral end of *Hoxd3* expression (Tümpel et al. 2009; Allen Developing Mouse Brain Atlas (http://developingmouse.brain-map.org/), whereas the r6/r7 boundary coincides with the rostralmost *Hoxd4* expression, and the caudal pole of the migrated facial motor nucleus. The facial efferents also serve as landmarks in this area; they first approach the periventricular genu across the r6 tegmentum; in the genu, the fibers ascend longitudinally through r5 (bypassing the abducens motor nucleus), and then course transversally into the nerve root within r4 (Figs. 1, 2 d, e).

At P10 we observed small-sized periventricular serotonergic cells in both r5 and r6, associated topographically to the facial genu, which we identified as *supragenual raphe* cell groups (r5SGeR and r6SGeR; Paxinos and Watson 2007) (Fig. 1a, b). There are also typical paramedian raphe cells at r5 and r6, as well as other serotonergic elements that are displaced laterally and superficially into a ventrolateral subpial area. We assigned the paramedian cells to the RMg complex, largely on the basis of shared gene expression patterns (see below). We distinguished therein dense ventral populations present at the superficial stratum—r5RMgV, r6RMgV—from rather dispersed dorsal populations found within the intermediate stratum—r5RMgD, r6RMgD (Figs. 1, 2d–g, 3a–c). Moreover, we identified the ventrolateral subpial cell groups as the parapyramidal raphe formation of the corresponding rhombomeres (r5PPy and r6PPy) (Figs. 1, 2f–h, 3f–h). See Table 1 for other terminologies.

##### R7 to r11 raphe nuclei

We tentatively located the rhombo-spinal boundary at a plane just caudal to the decussation of the pyramidal tract (xpy). The inferior olive complex extends between r8 and r11, appearing divided into two blocks corresponding to r8–r9 and r10–r11. The raphe obscurus (ROb) and raphe pallidus (RPa) nuclei extend rather uniformly along the paramedian basal plate of the caudal medulla from r7 to r11. RPa lies superficially, ventral to ROb that is restricted to the intermediate stratum. The ROb ends ventrally just dorsal to the inferior olive complex (IO), while the RPa is found more superficially, just dorsal to the pyramidal tract (py), along the midline that separates the right and left inferior olivary complexes (Figs. 1, 2h–m, 3a–c). Similar cells extend laterally from the RPa, encapsulating the pyramidal tract down to the pia lateral to it. These elements may be identified collectively as the medullary parapyramidal nucleus, with the corresponding segmental portions across r7–r11 (PPy) (Figs. 1, 2h–m, 3f–h). We also found some scattered median serotonin-immunoreactive cells caudal to the pyramidal decussation (xpy), that is, in the rostralmost spinal cord (Figs. 1, 2n). These may represent RPa cells dispersed caudalwards; they were more abundant at early developmental stages (see below). The most caudal neurons of the ROb are embedded in the pyramidal decussation (Fig. 2n).

##### Formation of raphe nuclei

Rostral cluster (Fig. 5): In the mouse, most serotonergic raphe neurons are born by E12.5. At that stage the rostral cluster is largely represented by three plurineuromeric pronuclei. One is the anlage of the DR, and consists of cells aggregated periventricularly next to the midline. This periventricular pronucleus starts immediately behind the midbrain–hindbrain boundary (MHB), where it is most dense, and becomes sparser caudalwards, stopping roughly at the boundary between rostral and caudal parts of r1 (Fig. 5a). It extends therefore across isthmus and rostral r1, clearly containing the primordia of the isDR and r1DR (note the mDR cells only start to be visible at the rostral end of this column at E14.5; Figs. 4, 5a, b). These nuclei are well formed by E18.5 (Fig. 5c). Only occasional serotonergic cells are found periventricularly at the levels of caudal r1 and r2, though a larger population appears in r3 (see below). Secondly, a separate paramedian population of serotonergic neurons is found more superficially at E12.5, adjacent to the median glial raphe, and partially approaching the pial surface, due to the momentary lack of the interpeduncular nucleus (Fig. 5a). This pronucleus starts at rostral r1 levels, and extends through caudal r1, r2 and r3 levels, representing a common primordium for the MnR, PPnR and r3PnR nuclei. A much sparser similar migrated population was observed within r4 (r4PnR; Fig. 5a, g). No such paramedian aggregate appears next to the midbrain (at the Is), though locally dispersed elements form a primordium of the CLi (Fig. 5a). At several levels across r1–r3, isolated cells appear at midcourse between the periventricular stratum and the MnR-PPnR-r3PnR pronucleus in the intermediate zone. Some serotonin neurons persist at such intermediate positions at later stages (E14.5, E18.5; Fig. 5b, c). It was characteristic of r2 that the PPnR elements extend continuously across the periventricular and intermediate strata. Finally, at E12.5 a separate ventrolateral pronucleus contains the future supralemniscal cells that migrate into positions close to the ventrolateral surface, apparently following a lateral radial course parallel to that of the larger paramedian migrated mass. The paramedian and ventrolateral migrated pronuclei are roughly coextensive rostrocaudally, but the ventrolateral cells are most abundant within r1, sharply decreasing in number more caudally. These three pronuclear groups do not change essentially at E14.5 and E18.5, irrespective of the sharpening of the individual components (Fig. 5).

**Fig. 6:**
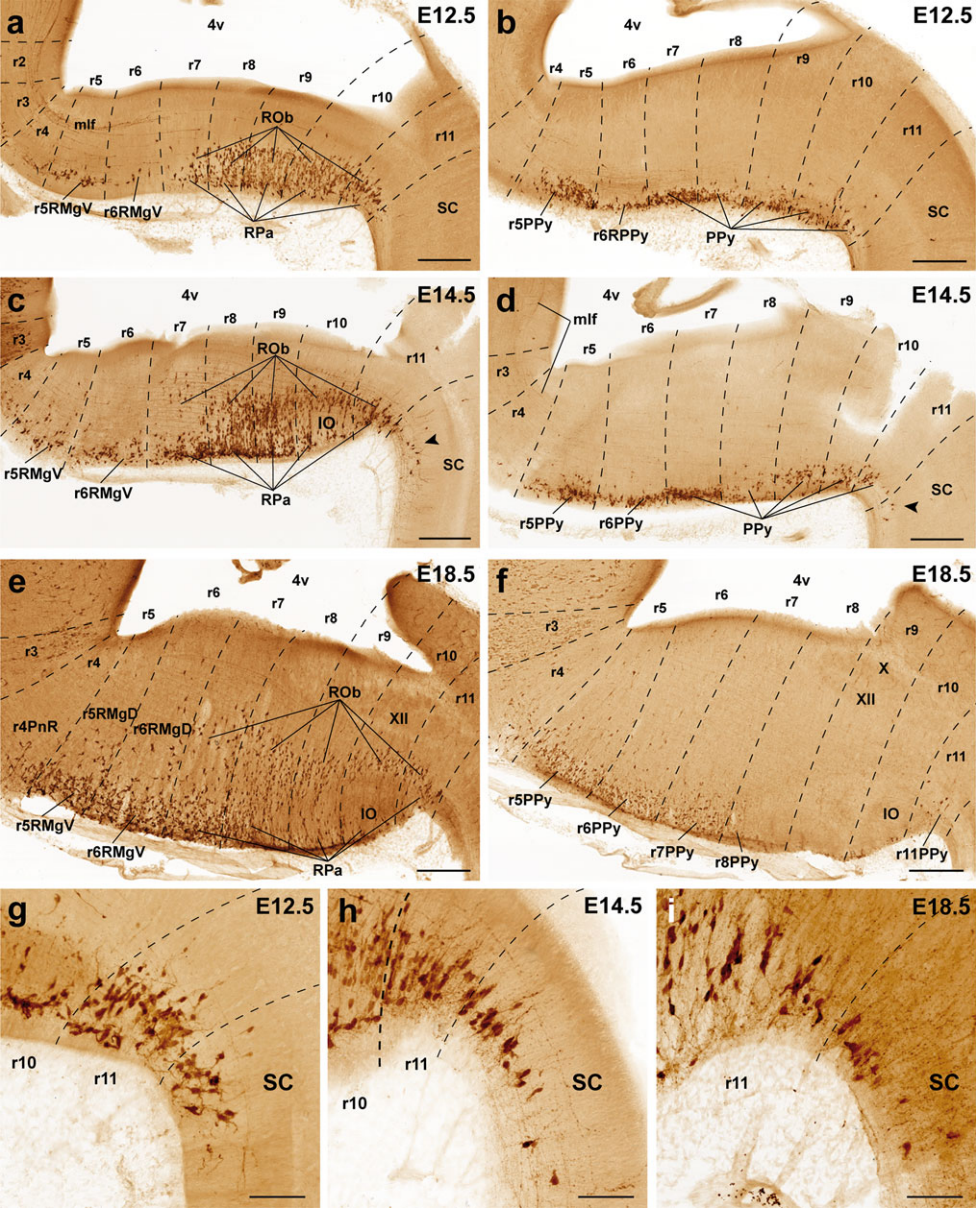
Segmental mapping of the caudal raphe cluster during embryonic development. 5-HT immunoreactive neurons observed in sagittal sections of mouse brains at E12.5 (**a**, **b**), E14.5 (**c**, **d**), and E18.5 (**e**, **f**), with superposed tracing of postulated interrhombomeric boundaries (*dashed lines*), and our tentative identification of the nuclear primordia (Table 1). Each set of three images read from top to bottom (e.g., **a**, **c**, **e**) represents a temporal sequence at a given section plane. **a**, **c**, **e** Findings at paramedian section level. **b, d**, **f** Findings at a more lateral sagittal section level. **g–i** Higher magnification of the rhombo-spinal boundary, showing presence of some 5-HT positive cells in the upper cervical spinal cord. Note that these cells are already present at E12.5 (see also *arrowheads* in **c** and **d**). For abbreviations see "List of abbreviations". *Scale bar* 300 μm in **a**–**e**, and 100 μm in **g**–**i**

Caudal cluster (Fig. 6): At E12.5, many serotonergic cells are already present ventrally next to the median glial raphe throughout the r5–r11 continuum, probably forming the primordia at least of the RMg and RPa. The ROb population may be added subsequently. Remarkably, there appears practically no periventricular differentiation of serotonergic cells in the pontomedullary and medullary domains. The small supragenual serotonergic cells only were observed postnatally, at P10 (Fig. 1). At the brain surface, the ventrolaterally displaced PPy pronucleus is established as well by E12.5 (Fig. 6b). These groups do not change essentially at E14.5 and E18.5 (Fig. 6).

##### Midbrain serotonergic cells

We corroborated the existence at P10 of a midbrain dorsal raphe cell group (mDR), which recently was attributed to the midbrain mesomere 2 (m2) by Puelles et al. (2012b). As opposed to the conventional idea that the whole DR complex lies in the midbrain, we thus confirm that only this small component lies rostral to the MHB, having now tested this point with the specific *Otx2* midbrain marker. The small m2 developmental unit was originally postulated by Palmgren (1921) and Vaage (1969, 1973), and resurfaced again in recent years due to molecular and experimental evidence shown in the chick by Hidalgo-Sánchez et al. (2005)—who called it ‘preisthmus’—and in the mouse by Puelles et al. (2012b); see also m2 in Martínez et al. (2012), Puelles et al. (2012a) and the abundant mouse genoarchitectonic evidence available in the Allen Developing Mouse Brain Atlas (http://www.developingmouse.brain-map.org).

**Fig. 7:**
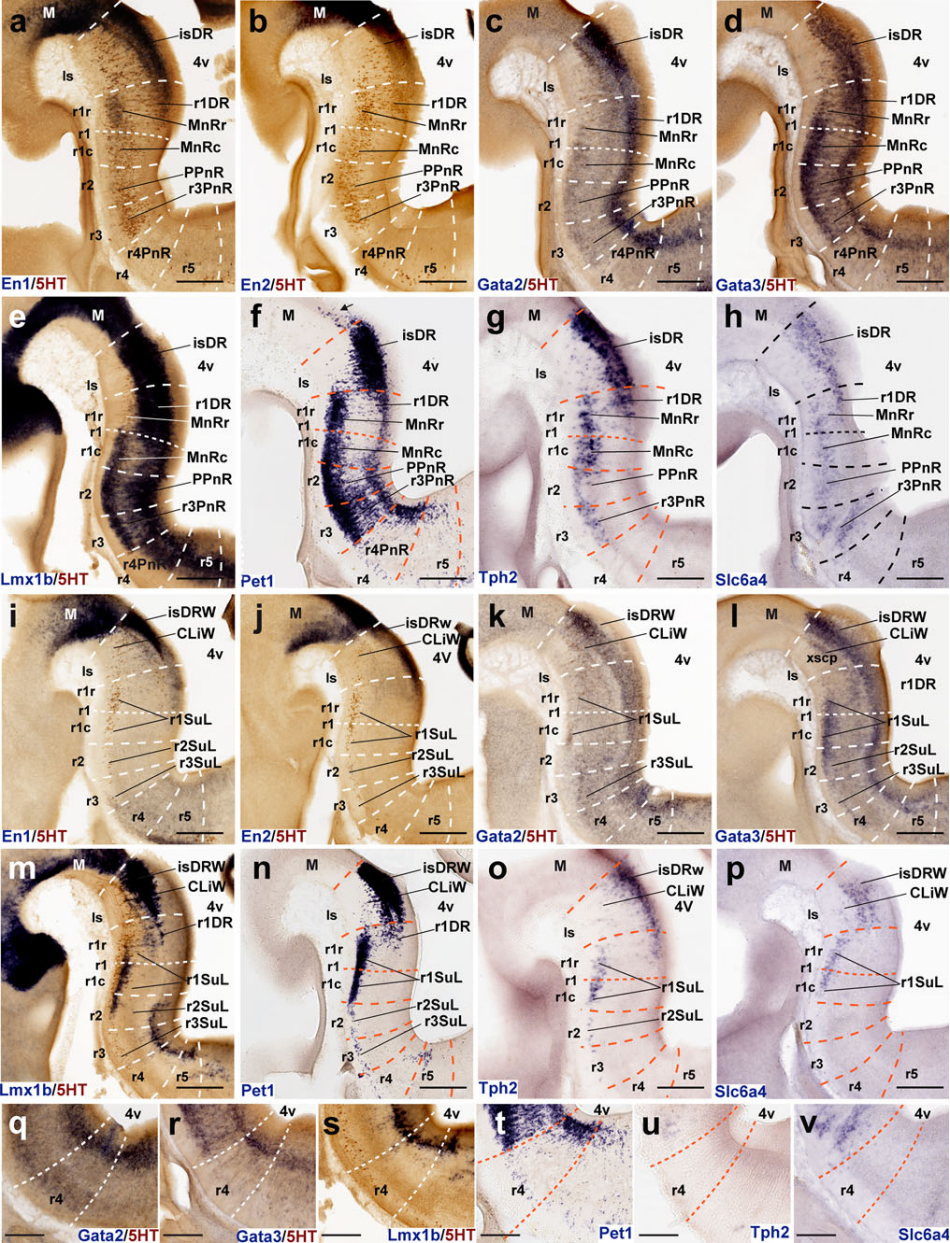
Expression of genes related with the serotonergic phenotype in the rostral raphe cluster in sagittal sections at E12.5. **a–h** Paramedian sections; the panels show 5-HT immunoreaction plus a particular in situ hybridization (**a–e**) or only an in situ hybridization (**f–h**). **i–p** More lateral section level; **i–m** show double 5-HT immunoreaction plus in situ hybridization, and **n–p** only in situ hybridization. **q–v** Higher magnification of r4 in six equivalent paramedian sections with 5-HT immunoreaction plus in situ hybridization (**q–s**), or only in situ hybridization (**t–v**). The riboprobes used are indicated in each case in *blue color* at the *lower left* (**a–q**) or lower right (**r–v**) corner. *Arrow* in **f** points to the mDR nucleus. *Scale bar* 250 μm in **a–p**, and 150 μm in **q–v**

Leaving apart a group of *Pet1*-positive cells observed at the same locus at E12.5 (Fig. 7f), serotonergic 5HT-immunoreactive neurons start to appear in the preisthmic midbrain at E14.5, shaped as a rostrally pointing spike connected with the isDR across the molecular M/R boundary delineated by *Otx2* (Fig. 4a, b, d, e, g, h). Their number increases significantly by E18.5 (Fig. 4c, f, i). Curiously, expression of *Otx2* seems reduced or even absent at the place occupied by these cells. The genetic profile at E14.5 of the mDR resembles that of its caudal neighbor, the isDR, which suggests that these neurons may migrate tangentially from the isthmus, where they would be born.

##### Genoarchitectonic labeling of raphe nuclei

A number of genes have been related to the differentiation of the serotonergic neurotransmitter phenotype. We chose eight of these markers and compared their expression patterns relative to our tentative map of rhombomeric units and the observed raphe nuclei primordia at E12.5, E14.5, E16.5 and E18.5 (E16.5 data not shown). We were interested in gene expression patterns indicative of either neuromeric heterogeneity (unique regional pattern) or metamery (repeated pattern). We also assessed molecular peculiarities accompanying the variable radial stratification of the diverse serotonergic populations. We mapped the transcription factors *En1*, *En2*, *Gata2*, *Gata3*, *Lmx1b*, and *Pet1*, known to act upstream of the serotonergic phenotype (among other roles), as well as *Tph2* (coding the rate-limiting enzyme that synthesizes serotonin) and *Slc6a4* (the solute carrier family 6 member 4, also known as the serotonin transporter). In addition, we examined and downloaded other data available in public databases (e.g., the Allen Developmental Mouse Brain Atlas). Our results revealed a common differentiation program of hindbrain serotonergic cells, with subtle neuromeric differences in the expression pattern of these genes and the stratification of the derived raphe populations, suggesting that a specific combination of signals applies to each rhombomeric raphe domain (summarized in Fig. 13).

For simplicity, we will present these data grouped into gene pairs mutually compared through the three developmental stages studied, dividing the material in two blocks dedicated to the rostral and caudal raphe clusters, respectively.

#### The rostral raphe cluster

##### *En1* and *En2*

**Fig. 8:**
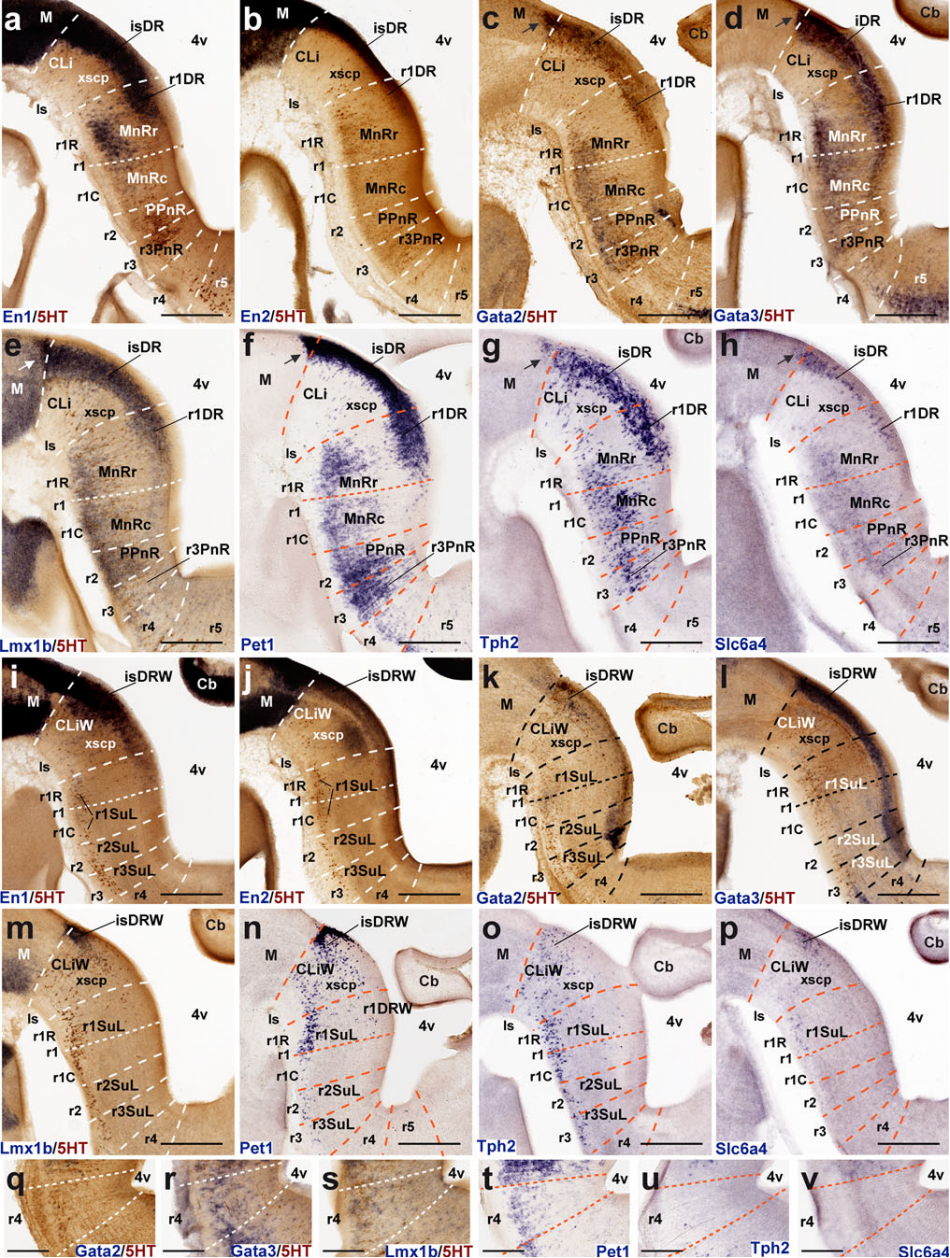
Expression of genes related with the serotonergic phenotype in the rostral raphe cluster in sagittal sections at E14.5. **a–h** Paramedian sections; the panels show 5-HT immunoreaction plus a particular in situ hybridization (**a–e**) or only an in situ hybridization (**f–h**). **i–p** More lateral section level; **i–m** show double 5-HT immunoreaction plus in situ hybridization, and **n–p** only in situ hybridization. **q–v** Higher magnification of r4 in six equivalent paramedian sections with 5-HT immunoreaction plus in situ hybridization (**q–s**), or only in situ hybridization (**t–v**). The riboprobes used are indicated in each case in *blue color* at the *lower left* (**a–q**) or *lower right* (**r–v**) corner. *Arrows* in **c–h** point to the mDR nucleus. *Scale bar* 250 μm in **a–p**, and 150 μm in **q–v**

**Fig. 9:**
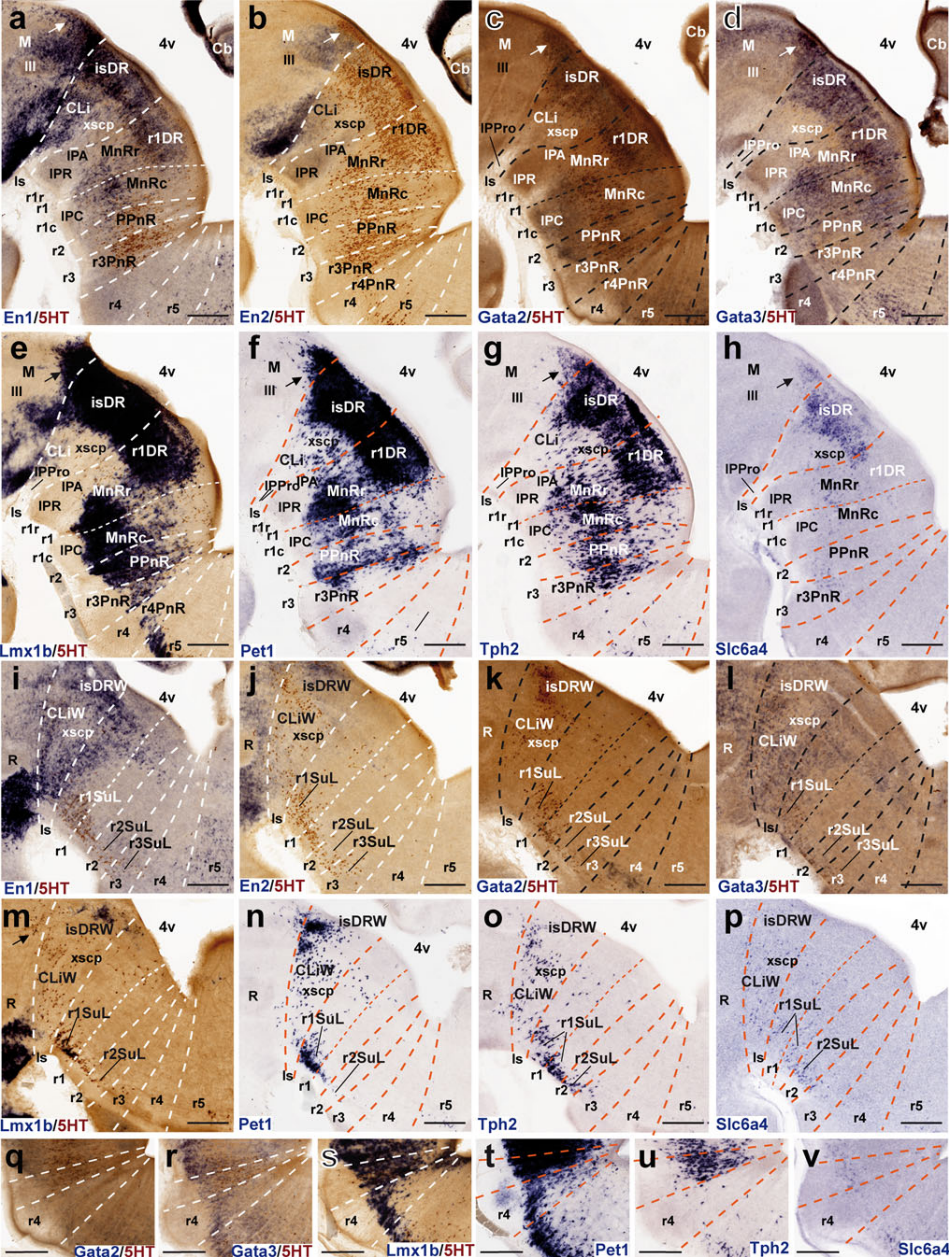
Expression of genes related with the serotonergic phenotype in the rostral raphe cluster in sagittal sections at E18.5. **a–h** Paramedian sections; the panels show 5-HT immunoreaction plus a particular in situ hybridization (**a–e**) or only an in situ hybridization (**f–h**). **i–p** More lateral section level; **i–m** show double 5-HT immunoreaction plus in situ hybridization, and **n–p** only in situ hybridization. **q–v** Detail of r4 in six equivalent paramedian sections (not identical with **a–h**) with 5-HT immunoreaction plus in situ hybridization (**q–s**), or only in situ hybridization (**t–v**). The riboprobes used are indicated in each case in *blue color* at the *lower left* (**a–q**) or *lower right* (**r–v**) corner. *Arrows* in **a–h** point to the mDR nucleus. *Scale bar* 250 μm

Both *En* genes were shown to play a role in the specification of some 5-HT-positive neurons of the rostral cluster (Simon et al. 2005; Wylie et al. 2010; Fox and Deneris 2012). Leaving apart their expression within the midbrain, the genes *En1* and *En2* are expressed in the rostral hindbrain in a decreasing gradient caudalwards from the isthmic organizer. *En1* and *En2* differ in spatial range of expression (*En1* more extensive than *En2*, particularly at E12.5; Fig. 7a, b; compare Fig. 9a, b) and in radial distribution of their expression. *En2* is largely restricted to ventricular cells at E12.5 and E14.5—absent at E18.5—while *En1* also appears in postmitotic neurons (Figs. 7a, b, 8a, b, 9a, b). At E12.5 and E14.5, *En1* is expressed by the periventricular DR pronucleus in a gradient across Is and rostral r1 (Figs. 7a, i, 8a, i). The derived isDR and r1DR nuclei are also positive at E18.5, the r1DR signal being weaker (Fig. 9a). As regards the intermediate paramedian and ventrolateral pronuclei, again only the CLi and MnRr paramedian cells (Is, r1r) showed strong *En1* signal at E12.5 and E14.5 (Figs. 6a, 7a). The MnRc (r1c) shows faint *En1* signal at E14.5; this labeling becomes more distinct at E18.5 (Fig. 9a). At the latter stage, the labeled CLi group appears more populated and is better separated from the DR complex (Fig. 9a). The DR wing portions and the r1SuL also show *En1* signal (Fig. 9i), but not so the r2 and r3 SuL analogs. Sparse *En1*-positive neurons were observed within the periventricular area of caudal r1, and a very low signal was detected at the PPnR in r2 (Fig. 9a). More caudally in the hindbrain other *En1*-positive neurons are observed, but they are not serotonergic (i.e., not immunoreactive for serotonin). At E18.5, ventricular *En2* expression largely has disappeared in medial sagittal sections, but persists at the isthmus more laterally, possibly indicating heterochronic regulation (Fig. 9b, j).

##### *Gata2* and *Gata3*

*Gata2* and *Gata3* are expressed in largely overlapping domains in the hindbrain. *Gata2* activates genes required for the specification of all 5-HT neurons (upstream of *Lmx1b* and *Pet1*), while *Gata3* function seems necessary only for the development of the caudal cell groups of the raphe (see “Discussion”). At E12.5, expression of both *Gata2* and *Gata3* is intense in the periventricular raphe pronucleus of the rostral cluster (but the positive column remarkably extends as far back as r6) (Fig. 7c, d, k, l). *Gata2* shows much weaker signal than *Gata3* at the incipient intermediate paramedian and ventrolateral pronuclei (Fig. 7c, d, k, l, q, r). At E14.5, *Gata2* expression diminishes to rather weak levels in the major part of the rostral cluster, and disappears completely within r4, though isolated cell groups are still positive at the immature DR and MnR nuclei (Fig. 8c, k, q). *Gata3* instead retains its expression at the periventricular and intermediate raphe pronuclei of the rostral cluster (the continuous positive periventricular stratum now stops at the r3/r4 limit; Fig. 8l); the ventrolateral superficial column (prospective SuL) does not show significant signal. Maximal *Gata3* signal appears at the DR and MnR formations (Fig. 8c, d, k, l). These differential patterns are even more evident at E18.5 (Fig. 9c, d, k, l). The periventricular positive cells found in r2 and r3 are not immunoreactive for serotonin (Fig. 9d).

##### *Lmx1b* and *Pet1*

**Fig. 10:**
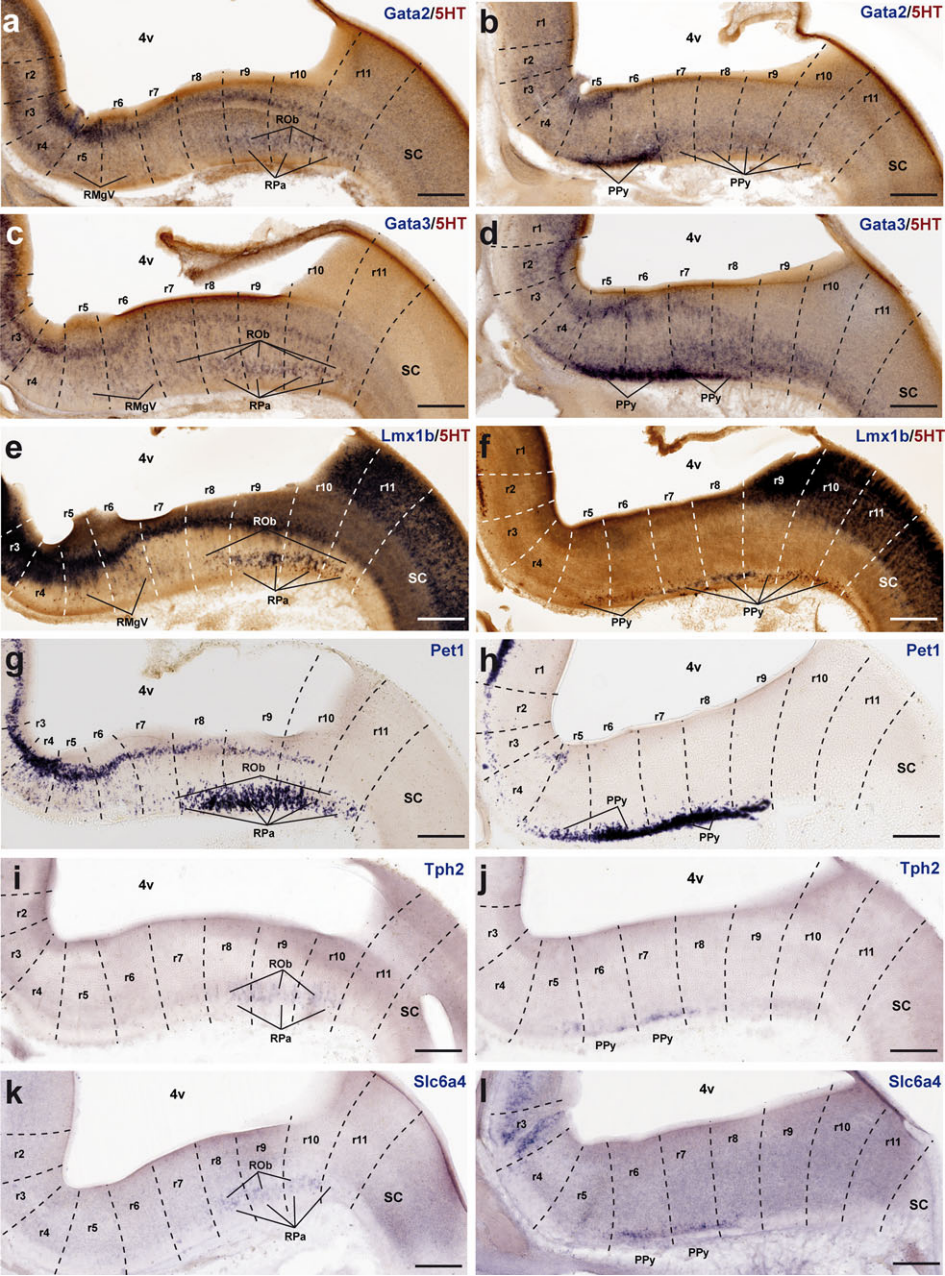
Expression of genes related with the serotonergic phenotype in the caudal raphe cluster in sagittal sections at E12.5. **a–l** Each pair of adjacent images represents paramedian and more lateral section levels reacted with a given probe and 5-HT immunoreaction (**a–f**) or only with an in situ probe (**g–l**). The relevant genes are indicated in *blue color* at the *upper right* corner of each photograph. *Scale bar* 250 μm

*Lmx1b* and *Pet1* are considered transcription factors linked to the differentiation of postmitotic 5-HT neurons; *Lmx1b* signal actually extends also within the basal midbrain and forebrain in association to dopaminergic cell populations (both progenitors and neurons; e.g., Fig. 7e, m), whereas *Pet1* is a specific marker of differentiated serotonergic neurons (Hendricks et al. 1999; Cheng et al. 2003; Ding et al. 2003). At E12.5, periventricular *Pet1* signal is strong in the isDR complex (Fig. 7f, m); the CLi and CLiW are not distinguished yet, and a few labeled cells appear within the neighboring midbrain periaqueductal gray (see arrowhead in Fig. 7f; note no serotonin-immunoreactive cells are found at this locus at this stage; Figs. 4g, 5a). The positive periventricular raphe cells are restricted to a sublayer of the periventricular pronucleus next to the ventricular cells themselves; this sublayer is much thinner at r1 levels and becomes again more populated more caudally, extending back at least to r6 levels (Figs. 7f, 10g). At r3 level a paramedian periventricular group of cells occupies the outer part of the periventricular stratum and appears partly connected with the local intermediate pronucleus (primordia of r3PnR). No such transitional cells exist at the isthmus, and few of them characterize the two parts of r1, or r2 (Fig. 7f). The intermediate raphe pronucleus formed medially across the r1–r3 continuum also expresses strongly *Pet1*. The isDRW and r1SuL cell groups are likewise strongly positive, though there are few labeled ventrolateral cells at r2 and r3 levels (Fig. 7n). In the hindbrain, *Lmx1b* seems to be expressed only in postmitotic neurons. However, it is readily apparent that *Lmx1b* labels the majority, if not all, of the periventricular postmitotic cells, thus labeling additional cells, apart those identified by *Pet1* and 5-HT-immunoreaction. This occurs not only across isthmus and r1–r3, but also continues caudalwards down to cryptorhombomere r10 (Figs. 7e, 10e). Other periventricular cell fates are clearly involved. The intermediate raphe pronucleus of the rostral cluster and the laterally displaced isDRW and ventrolateral groups are also positive for *Lmx1b* (Fig. 7e, m), though, remarkably, the rostralmost part of the intermediate column (prospective MnRr) apparently lacks altogether *Lmx1b* signal, as suggested by comparison of similar sections with *Pet1* ISH and immunoreaction for 5-HT (Fig. 7e, f, m, n). Other neuronal populations in the rostral hindbrain tegmental mantle (e.g., trochlear motoneurons and the interpeduncular nucleus) do not express *Lmx1b*. At E14.5, all periventricular populations caudal to the isthmus and r1r have lost the *Lmx1b* expression previously observed at E12.5 (Fig. 8e). Finally, the *Lmx1b* signal at E18.5 is readily comparable to that of *Pet1*, particularly as regards the DR complex, including its mesencephalic component (arrows in Fig. 9e, f) and the scanty periventricular cells in caudal r1 and r2 (Fig. 9e, f). The intermediate paramedian complex also shows massive labeling of MnR, PPnR and r3/r4 parts of PnR by both markers (Fig. 9e, f, t, u; note that our selection of comparable images of the DR complex causes that the other populations are not sectioned identically, coming from different specimens, giving the false impression that the intermediate nuclei are not equally labeled; the details in Fig. 9t, u illustrate this point).

##### *Tph2* and *Slc6a4*

*Tph2* and *Slc6a4* are genes expressed in differentiated 5-HT neurons. During development, expression of *Tph2* is delayed relative to that of *Pet1*. This is evident at E12.5; most signal is found at the Is and r1, at the DR, MnR and r1SuL primordia (Fig. 7g, o); levels caudal to r1 show little *Tph2* expression, both periventricularly and more superficially (Fig. 7g, o; compare 7f, n). Between E14.5 and E18.5, the *Tph2* pattern gradually approximates that of *Pet1*, but does not attain a comparable density (e.g., patchy signal in the periventricular stratum and sparse positive cells superficially; Figs. 8g, o and 9g, o). On the other hand, expression of *Slc6a4* in the same rostral raphe primordia is even more delayed relative to *Tph2* and *Pet1*. At the three stages examined, signal obtained with our *Slc6a4* probe was weak or moderate in the whole rostral cluster (Figs. 7h, p, 8h, p, 9h, p). Moreover, *Tph2* or *Slc6a4* cells were practically nonexistent within r4 (Figs. 7u, v, 8u, v, 9u, v), irrespective of the distinct *Pet1*-expressing and 5HT-immunoreactive cells mentioned above.

#### The caudal cluster

**Fig. 11:**
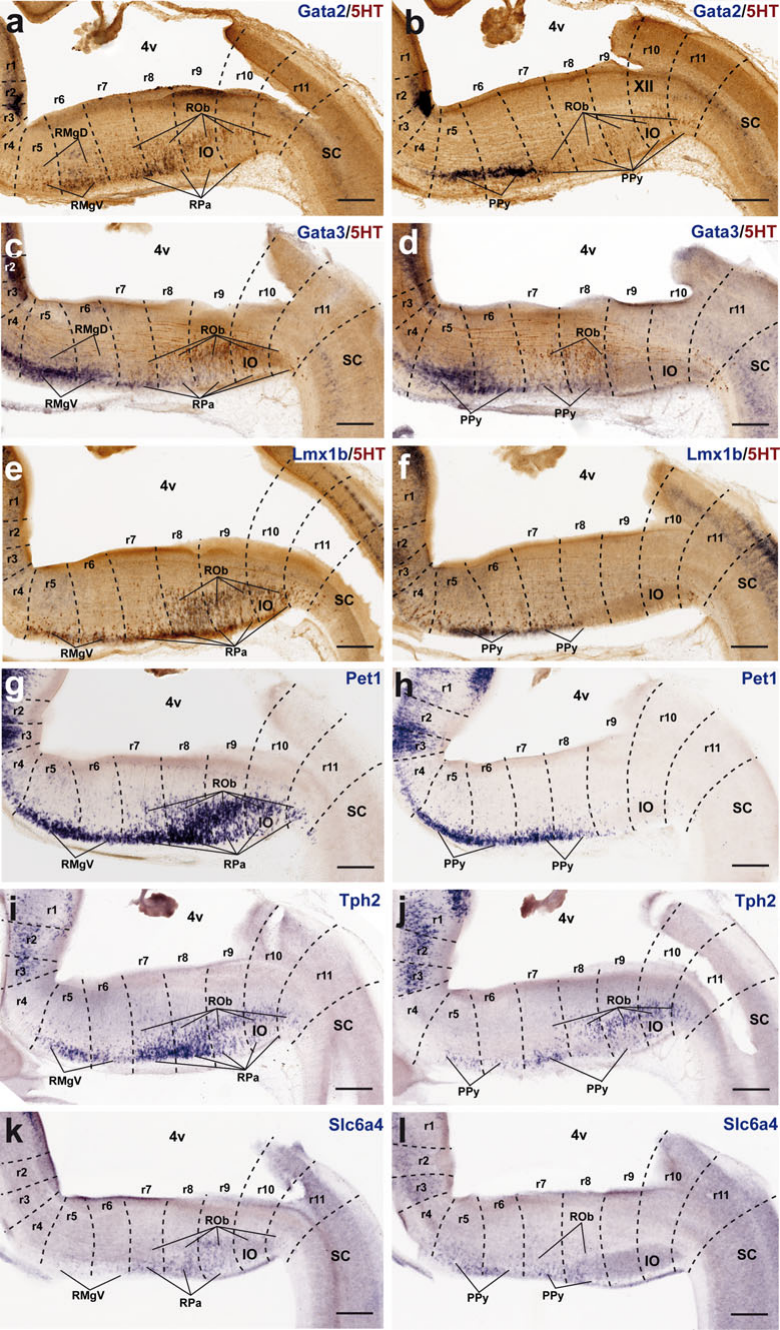
Expression of genes related with the serotonergic phenotype in the caudal raphe cluster in sagittal sections at E14.5. **a–l** Each pair of adjacent images represents paramedian and more lateral section levels reacted with a given probe and 5-HT immunoreaction (**a–f**) or only with an in situ probe (**g–l**). The relevant genes are indicated in *blue color* at the *upper right* corner of each photograph. *Scale bar* 250 μm

**Fig. 12:**
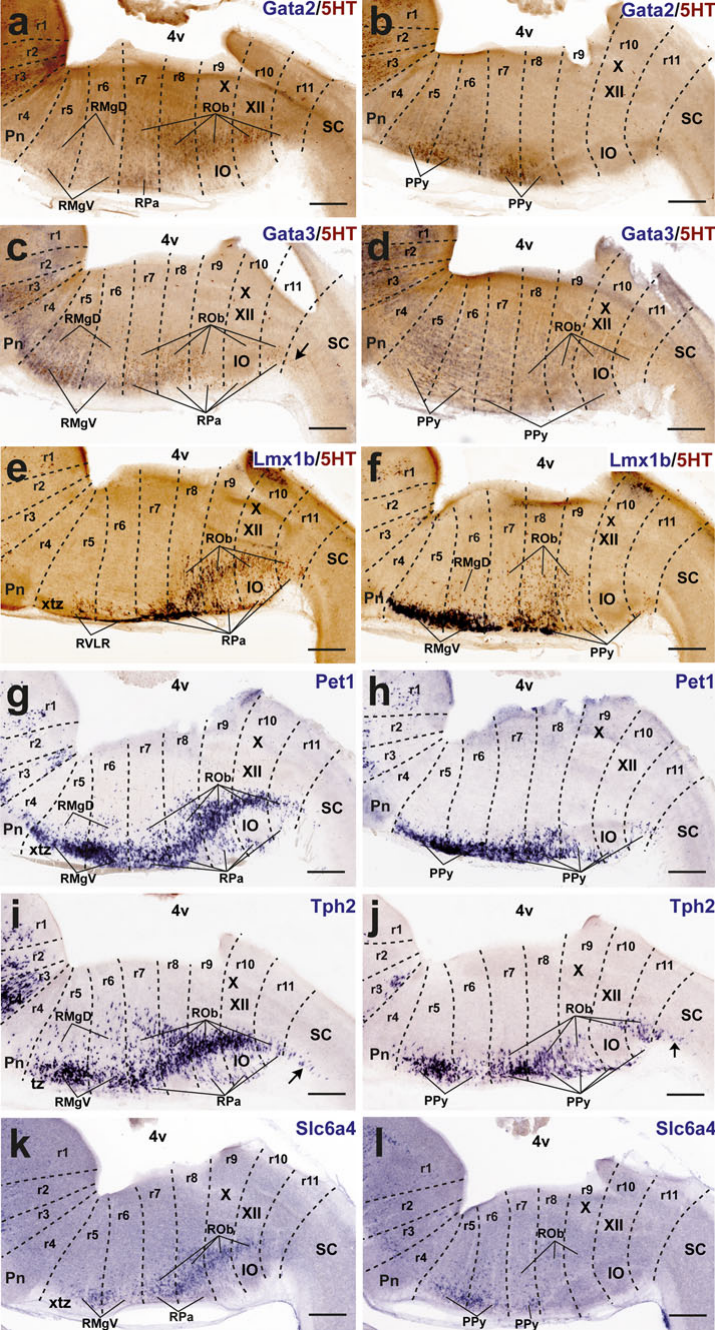
Expression of genes related with the serotonergic phenotype in the caudal raphe cluster in sagittal sections at E18.5. **a–l** Each pair of adjacent images represents paramedian and more lateral section levels reacted with a given probe and 5-HT immunoreaction (**a–f**) or only with an in situ probe (**g–l**). The relevant genes are indicated in *blue color* at the *upper right* corner of each photograph. *Scale bar* 300 μm

The P10 and adult caudal cluster of the raphe is characterized by including periventricular serotonergic cells within r5 and r6 (SGeR; Fig. 1). We did not detect them with any of the genoarchitectonic markers studied during prenatal development (they differentiate postnatally?). Nevertheless, the early embryonic periventricular stratum of the caudal raphe cluster does express transiently differentiation genes characteristic of the serotonergic lineage. At E12.5, this locus is characterized by either uniform or patchy expression of *Gata2*, *Lmx1b* and *Pet1* (continuing the patterns noted at the rostral cluster). These expression domains extend caudalwards at least into r10 (Fig. 10a, e, g). *Gata3* is expressed similarly, but more weakly (Fig. 10c). These pontomedullary and medullary periventricular populations decrease in importance, or disappear, at subsequent stages, either due to downregulation of the markers or depletion of the stratum via radial migration into the intermediate and superficial strata (Figs. 10a, c, e, g, 11a, c, e, g, 12a, c, e, g). However, some remnants persist at least until E18.5 (e.g., sparse periventricular *Lmx1b*-positive cells in Fig. 12f); such elements may be the precursors of the eventual supragenual raphe population at r5–r6. We next examine intermediate- and superficial-paramedian and parapyramidal raphe populations, emphasizing differences between the retropontine rhombomeres r5–r6 and cryptorhombomeres r7–r11.

##### *Gata2* and *Gata3*

At E12.5 *Gata2* and *Gata3* are generally weakly expressed in the intermediate stratum across the whole caudal cluster (Fig. 10a–d), whereas there is stronger expression at the superficial stratum, particularly in its ventrolateral (parapyramidal) part. The intermediate pronucleus gives rise to the RMgD in r5–r6 and the ROb in r7–r11 (Figs. 11a–d, 12a–d). *Gata2* signal predominates in the PPy primordium in r5 and r6, but is practically absent at the medullary parts of PPy (Fig. 10a, b). *Gata3* signal is even stronger at the r5/r6PPy, and extends also into r7 and r8 parts of PPy (Fig. 10c). None of them labels significantly the RMgV or RPa. At E14.5, *Gata2* and *Gata3* continue strongly expressed at the r5/r6PPy nucleus (Fig. 11b, d), and start to show signal at the RMgV (Fig. 11a–b). The medullary PPy primordium has relatively weaker *Gata3* signal (Fig. 11d). Weak paramedian superficial signal of the two markers appears in r7–r8, corresponding to rostral parts of the RPa primordium (Fig. 11a, c). At E18.5, expression of *Gata2* and *Gata3* has decreased significantly, particularly in r9–r11, while more rostrally the RMgV and PPy (r5–r6) as well as the RPa and medullary PPy populations (r7–r8) partially retain some *Gata3* expression (Fig. 12a–d).

##### *Lmx1b* and *Pet1*

At E12.5, *Lmx1b* is expressed in the r8–r10 subdivisions of the ROb/RPa pronuclei (Fig. 10e), while *Pet1* is intense in all groups of this caudal cluster, including cells in r11 (Fig. 10g). Expression of *Lmx1b* is rather weak and patchy at the RMgV and PPy, whereas *Pet1* appears intensely expressed at the PPy (Fig. 10h) and RMgV (not shown). At E14.5, a similar differential pattern of *Lmx1b* and *Pet1* is observed at the ROb/RPa (Fig. 11e, g). The RMgV and PPy nuclei express moderate levels of *Lmx1b* (Fig. 11e, f), whereas their *Pet1* signal is strong (Fig. 11g, h). At E18.5, *Lmx1b* signal, previously already absent at r11, seems now to have been wholly downregulated at r10, and is partly reduced at r9, where we detected many serotonin-positive cells negative for this gene. In contrast, its signal is still apparent at all pontomedullary and medullary raphe groups rostral to r9 (Fig. 12e, f). In comparison, *Pet1* expression is intense at the caudal cluster in general, specially at the superficial stratum of r6, where the RMgV and PPy appear very strongly labeled (Fig. 12g, h); subtle variations in the cell number and dispersion of the raphe populations correlate with cryptorhombomeric subdivisions (Fig. 12g, h).

##### *Tph2* and *Slc6a4*

Both genes are weakly expressed at the caudal complex at E12.5, their signal being barely detectable at the ROb/RPa primordia across r8–r10 (Fig. 10i, k) and at the ventrolateral PPy populations across r6–r8 (Fig. 10j, l). At E14.5 *Tph2* expression appears now strongly at the RMgV and ROb/RPa all the way to r11, but remains weak at the medullary PPy (Fig. 11i, j). In contrast, the *Slc6a4* signal remains weak throughout, an aspect that may be due to technical reasons (Fig. 11k, l). At E18.5, intense *Tph2* signal is now similarly distributed as *Pet1* signal (even extending slightly into the rostral spinal cord; arrow; Fig. 12i, j); the RMgV and PPy raphe groups in r6 are particularly distinct and other populations show subtle variations coinciding with postulated neuromeric subdivisions (Fig. 12i, j). *Slc6a4* expression has increased somewhat, but remains weaker than that of *Tph2,* and does not extend as far caudally (Fig. 12k, l).

### Genes of the Allen Brain Atlas database with restricted expression pattern in the raphe nuclei

We examined some genes from de Allen Brain Atlas database which showed restricted expression in the postnatal or adult raphe nuclei. We searched specifically for genes which are expressed differentially across the diverse serotonin populations identified in our analysis. It was expected that differences in regionally specific molecular background and/or terminal differentiation might be reflected in patterns characterizing selectively some nuclei across the major clusters, or superficial/lateral versus deep or intermediate paramedian serotonin cells across several rhombomeres. Here we show only a small selection of such genes, which indeed corroborate such differential molecular characteristics across the raphe system. Though the differential distribution is shown here optimally in paramedian sagittal sections, we corroborated in every case the implication that we deal with cells lying within true raphe nuclei in available coronal section material.

#### Genes expressed in the rostral and caudal clusters

We identified some examples of genes expressed in the two clusters, but restricted in both of them to specific cell subpopulations, clearly leaving other serotonin populations unlabeled: *Cbln2* (cerebellin 2 precursor protein), *Chrna7* (cholinergic receptor, nicotinic, alpha polypeptide 7), *Trh* (thyrotropin releasing hormone) and *Zfhx1b* (zinc finger homeobox 1b gene).

*Cbln2* is expressed in scattered mDR cells, separate ventral and dorsal subpopulations of isDR, fewer r1DRd/r1DRv cells, abundant rostral and caudal MnR elements, and scattered PPnR cells, largely excluding other parts of the rostral cluster (Fig. 14a). There were only sparse pontine positive cells. In the caudal cluster, *Cbln2* is expressed only in scattered r6RMg and in ROb cells, the latter extending caudally as far as r9, but there is no signal in RPa or in r5RMg (Fig. 14a).

*Chrna7* is expressed rostrally densely and very selectively at the r1DRd subdivision (no isDR or mDR) and in scattered MnR and PPnR cells, whereas caudally this gene is expressed in scattered cells at the RMgD and ROb, mainly across r5–r8 (Fig. 14b). R3 and r4 are devoid of any *Chrna7* signal.

*Trh* shows an even more restricted expression pattern, with few cells selectively present in the mDR, even fewer at the isDRd, and some detected at the r1SuL, whereas restricted cell patches decorate the r6RMgD, r7–r8RPa, and r10ROb (Fig. 14c), as well as the full PPy complex (not shown).

*Zfhx1b* is selectively and densely expressed at the isDRd subdivision (no mDR, and few r1DRd and r1DRv cells), as well as in scattered cells of the CLi, MnR, and PPnR. There are also some marked cells at the RMgV (r5 and r6) and r7RPa (Fig. 14d).

#### Genes expressed mainly in the rostral cluster

*Hdac6* (histone deacetylase 6) is preferentially expressed in the mDR, isDRd and isDRv, though there is also some dispersed signal at the MnR and PPnR groups (Fig. 14e). *Grm3* (glutamate receptor, metabotropic 3) labels strongly the inferior olive and basilar pons, and has weaker expression mainly restricted to the MnR, PPnR and PnR, although some positive cells appear as well at the mDR and isDR (Fig. 14f).

#### Genes mainly expressed in the caudal cluster

Given genes are expressed selectively at the RMg, or both RMg and ROb. For instance, *Cart* (cocaine and amphetamine regulated transcript) is expressed practically only in the r5–r6RMgD nuclei (Fig. 14g). In addition, *Esrrb* signal (estrogen related receptor, beta) is largely restricted to r5–r6RMgV (and nearby nucleus of the trapezoid body in r5), but appears as well in some PnR cells (Fig. 14h).

Other genes are medulla-selective. For instance, *Lhx3* (LIM homeobox protein 3) and *Npas1* (neuronal PAS domain protein 1) are two genes with a similar expression pattern restricted to intermediate paramedian serotonin cells of the caudal cluster. Both signals characterize RMgD in r5 and r6, as well as ROb in r7–r10 (Fig. 14i, j).

## Discussion

We examined the hypothesis already introduced by Cambronero (1999), Puelles et al. (2007) and Jensen et al. (2008) that serotonergic neuronal populations occupying raphe nuclei of the mouse hindbrain have a distinct neuromeric distribution, as well as some neuromere-specific molecular characteristics. Though we have concentrated on serotonergic neurons, it is well known that other neuronal types coexist at many of the raphe nuclei, as defined cytoarchitectonically (review in Nieuwenhuys 1985). In the present report, we studied the postnatal spatial distribution of 5-HT-immunoreactive and *Pet1*-expressing cell bodies relative to rhombomeric landmarks and the differential expression patterns of several genes involved in serotonergic differentiation at given developmental stages. A number of points relative to our criteria for performing the segmental mappings were argued in “Results”. Some extrapolation and, therefore, some inherent error, are implicit in our procedure. Our findings about a raphe segmental pattern in the mouse are largely consistent with similar earlier analyses done in the chick (Cambronero 1999; Puelles et al. 2007) or mouse (Jensen et al. 2008). The observed pattern is probably conserved at least in tetrapods, if not shared by all vertebrates (Nieuwenhuys 1985, 2009). In the following section we will compare this emerging segmental scenario of serotonergic raphe neuronal populations with previous models of this neuronal system, which uniformly assumed concepts now held to be obsolete about anteroposterior hindbrain boundaries. An additional source of confusion was the unfortunate convention to accept standard atlas coronal sections through rostral hindbrain as being transversal, when they really are horizontal (see Fig. 2o; e.g., the so-called *dorsal* raphe nucleus is actually a *rostral* structure). We will also examine how this map correlates with reported raphe heterogeneities regarding cellular morphology, neurochemical profile and patterns of connectivity. Conceptually, a segmental model of the raphe system leads us to expect heterogeneities, since rhombomeres have partially distinct molecular identities that might cause variant phenotypic aspects, as opposed to the common neurotransmitter phenotype, which is probably due to a set of shared genetic determinants.

### Old and modern views on brainstem boundaries bear upon our topographic conception of the raphe nuclei

In general, available descriptions of the distribution of murine raphe nuclei are based on the traditional anatomic model of the midbrain and hindbrain regions in mammals, which is strongly laden with concepts taken from human brainstem anatomy. The basic subdivisions considered were the midbrain, the pons and the medulla, and, accordingly, raphe populations were assigned in mammals to mesencephalic, pontine or medullary territories (e.g., Taber et al. 1960; Dahlström and Fuxe 1964; Swanson 1992, 1998, 2003; Paxinos and Franklin 2007; Dong and The Allen Institute for Brain Science 2008; see Fig. 15b). This pons-dominated conception is now thought to need drastic corrections, due to its lack of a consistent fundament in developmental data (Puelles et al. 2007, 2012b; Watson and Paxinos 2010; Watson et al. 2010; Martínez et al. 2012; Watson 2012). Only rough anatomic criteria were available some 100 years ago for delimiting rostrally and caudally the adult pons (e.g., the ponto-mesencephalic and ponto-medullary surface sulci, due to relief of the pontocerebellar fibers of the middle cerebellar peduncle). Even these simple landmarks were liable to cause confusion, particularly when applying what is valid for human anatomy to rodent anatomy. Indeed, it passed generally unnoticed that in rodents all pontocerebellar fiber course rostral to the trigeminal root, so that there is no ponto-medullary sulcus analogue; this situation changed in primates and other large mammals with further evolutionary development of the pons and the cerebellum (Nieuwenhuys 2001, 2009). Moreover, the mammalian basilar pons and its cerebellopetal fibers are essentially added superficial structures relative to the more primitive hindbrain tegmentum within (via evolution of the rhombic-lip-derived tangential pontine migration), so that the extrapolation of apparent pontine limits into the depth of the brainstem is not conceptually solid (or developmentally consistent). The simplistic subdivision of the hindbrain into pons and medulla was practical for the status of neuroanatomy 100 years ago, but was suspect already at that time; it essentially disregarded antecedent developmental data on a prepontine or isthmic component of the hindbrain, present also in humans (His 1893, 1895), as well as accrued data on hindbrain segmentation in all vertebrates (note that discovery of rhombomeres at the late nineteenth century—e.g., Orr 1887—preceded the discovery of raphe nuclei; see reviews in Vaage 1969, 1973; recent updates appear in Nieuwenhuys 2009, 2011; Watson 2012; Puelles 2012, in press).

These long-accepted, conventional ‘pontine’ limits for the human midbrain and medulla, which have been the basis for raphe nuclei classification, are no longer satisfactory from our present-day perspective, since they clearly do not agree either with the modern molecularly defined isthmo-mesencephalic border (e.g., Zervas et al. 2004; Jensen et al. 2008) and the related, now firmly established, critical patterning role of the isthmic organizer, nor with any of the developmental boundaries separating the now widely accepted rhombomeric histogenetic units.

The number of such units considered here may need comment. The overtly bulging r1–r6 units, or overt rhombomeres (delimited early on by outer constrictions), were long thought to be transient (and therefore unimportant for anatomic or functional explanatory purposes), but we now know by fate-mapping that they actually only become modified by thickening of their walls as the mantle layer differentiates, thus losing the limiting interrhombomeric constrictions, but their derivatives in the mantle maintain the primary intrinsic boundaries (fate maps by Marín and Puelles 1995; Wingate and Lumsden 1996), as well as the corresponding molecular coding differences (Marín et al. 2008). Other parts of the hindbrain found rostral and caudal to the set of overt rhombomeres never show transverse delimiting constrictions, but comparable cryptic (hidden) boundaries have been found, identifiable in the embryonic and mature hindbrain as fate limits and molecular limits; this is the case of the so-called crypto-rhombomeres Is and r7–r11 (Cambronero and Puelles 2000; Marín et al. 2008, Lorente-Cánovas et al. 2012). Note there are authors that do not distinguish Is from r1 proper (for instance, Jensen et al. 2008); they obviously allude as well to isthmic derivatives each time they refer to ‘r1’.

These results on the whole corroborate the idea that the causally important boundaries are the intrinsic ones, due to molecular patterning, since these condition differential histogenesis and anatomic structure. Surface constrictions are epiphenomena of morphogenesis, and may be visible counterparts of true molecular limits or not. From that point of view, crypto-rhombomeres are true rhombomeres. A further point in the background of our approach is the circumstance that the roots of the cranial nerves generally show invariant positions relative to the set of 12 rhombomeres, irrespective of the amount of axial bending that the hindbrain may suffer during morphogenesis. A preliminary discussion of the issue of anatomic discrepancies emerging with modern molecular analysis of hindbrain boundaries, and touching on the morphologic meaning of the cranial nerve roots, appeared in Rubenstein and Puelles (1992).

We will deal now with some current errors inherited from the recent 100 years of non-segmental neuroanatomy. This era began at the start of the 20th century with the discovery by Gaskell (1889) and Johnston (1902) of hindbrain columns; general enthusiasm about their functional importance had the effect of relegating apparently non-functional and supposedly transient rhombomeres to oblivion. Nevertheless, rhombomeres have returned as important patterning and histogenetic units with the molecular era, being remarkably consistent with multiple results from transgenic progeny tracing, mutated phenotypes and genoarchitectony.

A relevant point bearing on the classification of rostral raphe nuclei is the definition of the midbrain–hindbrain boundary (MHB; Zervas et al. 2004; see also Puelles et al. 2012b), since these formations have been systematically misclassified as being mesencephalic as a whole. The MHB lies at the midbrain–isthmus interface (originally identified by His 1893, 1895, and corroborated by Palmgren 1921 and Vaage 1969, 1973), where the isthmic organizer exerts a long-range inductive influence in both directions (the boundary itself is marked by apposed thin transverse rings of *Wnt1* and *Fgf8* expression, and by the apposition of wider fields of expression of *Otx2* and *Gbx2;* Hidalgo-Sánchez et al. 1999, 2005; Simeone 2000). These findings on rostral hindbrain patterning, which included the mechanism for the development of the cerebellum, first emerged in the late 1980s (Martinez and Alvarado-Mallart 1989). It is now strongly supported by a variety of experimental studies and several mouse mutant phenotypes, corroborating the existence and complexity of a sizeable rostral portion of the hindbrain that is essentially prepontine in developmental topography and causal background. A portion of the interpeduncular fossa and peduncles lying caudal to the oculomotor root, e.g., at the locus of the interpeduncular nucleus, as well as the whole pedunculopontine, isthmic and parabrachial areas, belong to the rostral hindbrain, and must be interpreted, together with the contained raphe nuclei, as Is, r1 and r2 derivatives. The midbrain accordingly does not contact the pons at all, and, as we have seen, only a minor rostral part of the dorsal raphe nucleus can be attributed to the caudal midbrain (Puelles et al. 2012b; Allen Developing Mouse Brain Atlas; http://www.developingmouse.brain-maps.org).

Note that the basilar pontine nuclei selectively aggregate within r3 and r4 after their tangential migration (they originate at the rhombic lip roughly at r6–r7 levels). Similarly, the transitional pontomedullary domain (r5–r6) separates the pons from the hindbrain medulla proper (r7–r11), most of which is characterized by the inferior olive. The basilar pontine nuclei in r3–r4 therefore can be precisely delimited developmentally from the set of prepontine hindbrain histogenetic units (isthmus, plus r1 and r2), as well as from the retropontine ones (r5–r11). This pattern is common to all vertebrates and should be the fundament of hindbrain anatomy.

It is also relevant to consider the developmental position of the cerebellum, due to the classic notion of a pontocerebellar developmental unit, which has turned out to be fictitious. The cerebellar vermis derives from the Is, and the cerebellar hemispheres and flocculus from r1 (see our Figs. 2, 3; review in Hallonet and Alvarado-Mallart 1997). In any case, the entire cerebellum clearly develops rostral to the pons (in r3, r4) in a dorsal prepontine hindbrain domain subject to the inductive influence of the isthmic organizer, whereas the pons proper develops outside of that domain. Therefore, the cerebellum is not a dorsal appendage of the pons other than topographically, irrespective of what is taught under traditional anatomic assumptions. Consequently, the middle cerebellar peduncle is not a transversal or ventrodorsal pathway. In the classic anatomic conception, the isthmus and extracerebellar r1 domains were wrongly included either in the pons or in the midbrain, and r2 jointly with the entire cerebellum was systematically assigned to the pons. This last error was probably caused by the fact that ponto-cerebellar fibers (all of them in rodents and most small mammals) course into the cerebellum via r2 and r1, passing first longitudinally rostral to the trigeminal root, which always enters the brainstem at the caudal end of r2, before bending into the cerebellum. Only primates, cetacea and other large mammals have ponto-cerebellar fibers coursing behind the trigeminal root (Nieuwenhuys 2001, 2009). As a result, the trigeminal root is widely described as entering the ‘pons’, although sectioned material will show that this root only relates to the *brachium pontis*, rather than to the pontine nuclei. Unfortunately, it was not thought necessary classically to distinguish the ‘pons’ sensu stricto, that is, the basilar pontine nuclei in r3–r4, from the ‘pons’ sensu lato, which includes the pontocerebellar fibers, coursing via r2 into r1 (primitively) or also via r3 and r4 into r2 and then r1 (in primates and large mammals).

The modern pontomedullary boundary (PMB), defined just caudal to the basilar pontine nuclei, lies between r4 and r5, ventrally separating macroscopically the basilar pons from the trapezoid body (Fig. 15c). This contrasts with the classical concept, in which part of the retropontine r5 and r6 developmental units (containing the abducens and the migrated facial motor nuclei, as well as the trapezoid body and superior olivary complexes), were wrongly assigned to the ‘pons’, at least in human neuroanatomy textbooks. The raphe magnus nucleus falls into this domain. This error apparently was due to the ventral bulge of the much deformed r4 basilar pontine region in the human brainstem, which sags like an apron over the ventral surface of r5 and r6 (this does not occur in rodents; Nieuwenhuys 2001, 2009). Notably, the abducens nerve arising at r5 level always emerges freely from under the pons (Nieuwenhuys 2001). The caudalmost cerebellopetal pontine fibers, being oriented toward the cerebellum in r1, do not cover the dorsalmost parts of r2–r4. This is demonstrated by the fact that the facial and cochleo-vestibular nerve roots, which penetrate the hindbrain through r4, and therefore are proper pontine nerves, do not traverse the pontocerebellar fibers, but enter the free pial surface of the r4 alar plate dorsocaudal to the *brachium pontis*; similarly, the cochlear nuclei formed next to the rhombic lip in r2–r5 lie at the brain surface, free of pontine fibers.

This developmental topologic analysis leads to the conclusion that the two macroscopic sulcal boundaries of the pons, which delimit the middle cerebellar peduncle, are not really transversal anatomic boundaries relative to the true developmental units, the rhombomeres, and even are not constant in mammals. Accordingly, they do not coincide with the causally relevant transversal neuromeric molecular boundaries. Moreover, the midbrain is separated from the pons by a sizeable prepontine hindbrain domain (Fig. 15c; this conception is represented in the Allen Developing Mouse Brain Atlas). Modern molecular and causal understanding of hindbrain structure thus requires us to downplay the relative importance of the classic pons concept, and we have to accept the caudal limit of the midbrain in front of the isthmus, the prepontine nature of cerebellum, isthmus, r1, and r2, and a retropontine pontomedullary region (r5–r6) that holds the trapezoid body and the facial motor nucleus. The medulla concept itself would be restricted to r7–r11, wherein the inferior olive appears at r8–r11 levels. The rhombo-spinal boundary lies developmentally across the fifth somite (fate mapping by Cambronero and Puelles, 2000), which corresponds to a plane just caudal to the pyramidal decussation (in r11).

The conserved topography of the entrance points and intraneural courses of the cranial nerve roots relative to rhombomeres in all vertebrates (Vaage 1969; Nieuwenhuys 1998, 2009) provides the strongest help for recognizing in sagittal and horizontal sections the mature derivatives of the relevant hindbrain histogenetic fields, as has been corroborated by a number of modern experimental or transgenic fate-mapping studies (e.g., Carpenter et al. 1993; Marín and Puelles 1995; Studer et al. 1996; Gavalas et al. 1997; Schneider-Maunoury et al. 1998; Cambronero and Puelles 2000; Oury et al. 2006; Tümpel et al. 2009). This is the novel background for our segmental analysis of the raphe nuclei.

### Neuromeric topography and classification of the raphe nuclei

Complementarily to the partial and somewhat simplistic treatment offered by Jensen et al. (2008), our present map of raphe nuclei redresses the mentioned descriptive inaccuracies due to the traditional anatomic model, by taking into account the topological relations of all serotonergic neuronal populations found within the 12 rhombomere-derived domains (isthmus plus r1–r11), thus offering for the first time a coherent full explanation of their evident heterogeneity along the longitudinal axis (Figs. 1 and 15). Beyond the existence of 12 separate rhombomeric origins, the variety of raphe populations is increased by the fact that serotonergic neurons developing within each rhombomere may adopt diverse stereotypic radial and/or medio-lateral positions within the basal plate. Note that, irrespective of their collective name identifying them as ‘raphe’ formations, none of the studied cell groups originates from the raphe proper, which is a *Shh*-positive median astroglial palisade formed at the hindbrain floor plate. Serotonergic raphe neurons are generated instead from an adjacent *Nkx2.2*-positive strip (formed in response to local strong Shh signaling; Shimamura et al. 1995), which constitutes the paramedian or ventralmost microzone of the hindbrain basal plate (Briscoe et al. 1999). The raphe populations normally migrate radially into the local mantle, and differentiate there, occupying, therefore, a paramedian position adjacent to the glial raphe, either periventricularly or within the intermediate or superficial strata. Secondary migratory invasion of the median raphe territory by some serotonergic neurons occurs at later developmental stages, particularly in the dorsal raphe complex. Other raphe populations migrate in the opposed, lateral direction, reaching slightly more lateral positions at any of the three strata. A majority of these laterally displaced cells aggregate superficially (e.g., PPy cells), though others are secondarily separated from the pial surface by some later developing structure (usually tracts, or the basilar pons); the latter cells are found postnatally in ventrolateral parts of the basal plate territory (e.g., the SuL cells; Lidov and Molliver 1982; Wallace and Lauder 1983; Goto and Sano 1984).

Based on the tridimensional distribution of serotonergic neurons, we conclude that there exist at least forty-five distinct raphe nuclei, leaving apart dispersed cells in-between. Since often there are similarities in their radial and mediolateral location across some neighboring rhombomeres (possible shared guidance mechanisms, or adhesive properties), the individual periventricular, intermediate, lateral and ventrolateral (superficial) nuclei can be grouped into a number of plurineuromeric nuclear complexes, which largely correspond to the serotonergic cell groups conventionally recognized in the classic and alpha-numeric terminologies (Table 1). From a developmental perspective, rostral (prepontine and pontine) and caudal (pontomedullary and medullary) developmental clusters were already identified previously (Olson and Seiger 1972; Seiger and Olson 1973; Lidov and Molliver 1982; Wallace and Lauder 1983; Goto and Sano 1984).

The segmental character of the rostral cluster was revealed by Jensen et al. (2008); these authors used sophisticated intersectional and subtractive genetic fate-mapping tools to investigate postnatal serotonergic cells (marked by expression of *Pet1*) that were derived, respectively, from what the authors identified as ‘r1’ (the sum of Is and r1; selected by the co-expression of *En1* in this territory), r2 (by co-expression of *Rse2*) or r3 plus r5 (by co-expression of *Egr2*). The labeled rostral hindbrain raphe domains identified in this way essentially correspond to our results, particularly in underlining the contribution of Is and r1 (not distinguished in this study) to the DR complex (B7, B6), but jointly also to the rostral MnR (part of B8) and SuL elements (part of B9). The existence of a mDR component was not identified (the ‘r1’ labeling via *En1* could not have distinguished the possible difference between midbrain versus isthmic origins, in any case). Our present analysis goes one step further in distinguishing midbrain, isthmic and r1 parts within the ‘r1’-derived complex, allowing finer analysis of the DR complex (needed, as we showed, by some available genoarchitectonic labeling patterns). Other populations that had conventionally been attributed to caudal parts of B8 and B9, were shown to be derived from r2 and r3; these clearly include our caudal MnR, PPnR and r3PnR cell groups, as well as the corresponding SuL groups (the pontine nature of some of these neurons was not mentioned). The serotonergic derivatives of r5 were deduced to include the RMg (its rostral half, according to us; see also Bang et al. 2012). No serotonergic cells were assumed a priori to derive from r4 (a dogma in the literature), and, consequently, it was not determined whether any pontine serotonergic cells were left unlabeled by either r3 or r5 fate mapping. The sketched median projection of segmental raphe populations offered by these authors (in their Figure 2a) is remarkable by the implied enormous size of the r4 domain, compared for instance with r2, r3 and r5 (compare r3 versus r4 in our Fig. 1c). This may be due to artistic license. Possibly similar data shown on sagittal sections would have demonstrated even closer correspondence with our mappings. An important point made by Jensen et al. (2008) is that there is some intermixing of raphe cells derived from adjacent rhombomeres (corroborating similar general conclusions of Marín and Puelles 1995; Wingate and Lumsden 1996; Cambronero and Puelles 2000; Marín et al. 2008). Finally, Jensen et al. (2008) also concluded that some of the ‘r1’ derivatives aggregate caudally at the supragenual or B4 cell group; this would necessarily imply a tangential migration of ‘r1’ elements into r5–r6, for which we did not see any support in our developmental analysis. We think that this discrepancy probably can be explained by assuming that their supposed B4 cells actually represent the sparser r1DRv cells found by us (also by Hale and Lowry 2011) periventricularly at caudal r1 level (that is, they would need to be interpreted as caudal B6, rather than B4; this would eliminate the need of conjecturing a very odd migration); the relevant illustrated material in cross sections in Jensen et al.’s (2008) Figure 2 does not show convincingly the genu of the facial nerve, which is a required landmark for the supragenual B4 cells.

Rhombomeric groupings are useful for the purpose of simplifying the terminology, but do not presuppose functional identity of the individual segmental components (the contrary is true, since different segmental origins, involving varying molecular identities, raise the possibility of subtle structural differentiations and corresponding functional consequences). We will discuss below some hodological peculiarities. Accordingly, we hold that any proposal of functional unity across a plurineuromeric raphe aggregate would need to be demonstrated experimentally, irrespective of the superficial anatomic similarity. The latter may be due merely to shared cell-positioning mechanisms.

In our segmentally adapted nomenclature, we tried to conserve as far as possible the conventional denominations of the raphe nuclear complexes (e.g., DR, ROb, RPa; Olszewski and Baxter 1954; RMg found in Taber et al. 1960; MnR found in Dahlström and Fuxe 1964; the same approach can be applied to the B group names of the alternative alpha-numeric terminology; see Fig. 15 and Table 1). The names ‘supralemniscal’ and ‘parapyramidal’ used by us are already found in the literature (Table 1). We followed the logic that apparent plurineuromeric sharing of radial and mediolateral topography across several rhombomeres is due to similar histogenetic mechanisms, irrespective of potentially variant individual molecular identities of their segmental units; a shared name thus seems also apposite.

#### The rostral cluster

The classic rostral cluster is essentially isthmic, prepontine and pontine, and contains diverse parts of the dorsal (DR), median (MnR), prepontine (PPnR) and pontine (PnR) raphe nuclei, apart supralemniscal ventrolaterally placed cells (Puelles et al. 2007; Jensen et al. 2008; present results). We should remember that, developmentally, the mesencephalic, isthmic and r1 elements arise within the area of influence of the isthmic organizer, whereas the prepontine and pontine ones lie outside it.

We found that the hindbrain DR complex lies mainly across the isthmus and rostral half of r1, with a tardive minor extension into the caudal or preisthmic midbrain (m2). We deal separately below with the mesencephalic component of the DR complex. Remarkably, the entire DR complex, or most of it, is conventionally wrongly thought to be mesencephalic, due to the historic reasons sketched above (e.g., Swanson 1992; Paxinos and Franklin 2007; Dong and The Allen Institute for Brain Science 2008), while Jensen et al. (2008) and Bang et al. (2012) interpret it entirely as rhombencephalic. This point was specifically reexamined here by comparison of the developing DR with the selective midbrain marker *Otx2*. The latter expression domain clearly stops just in front of the DR complex up to E14.5, when the minor midbrain component starts to appear.

There are morphological and neurochemical antecedents of the m2, isthmic and r1 segmental subdivisions of the DR deduced by us. Several authors recognized three anteroposterior parts of the DR, based on distinctive patterns of cellular distribution and morphology (Dahlström and Fuxe 1964; Daszuta and Portalier 1985; Ishimura et al. 1988; Eaton et al. 1993; Abrams et al. 2004; Fu et al. 2010): the rostral and caudal portions (our mDR and r1DR, respectively) were found to be restricted to the midline, while the intermediate portion typically shows wing-like lateral expansions (our isDR with its lateral ‘wings’; Hale and Lowry 2011). Topographic mappings of neurotransmitters and neuropeptides in the DR nuclear complex are also consistent with our three subdivisions: TH (tyrosine hydroxylase), somatostatin and CCK-(cholecystokinin) expressing neurons are present in the mDR (van der Kooy et al. 1981; Vanderhaeghen 1985; Priestley et al. 1993; Smith et al. 1994; Araneda et al. 1999; Fu et al. 2010; Puelles et al. 2012b). The isDR selectively contains a cell population positive for GAD67 (Fu et al. 2010), and enkephalin, NOS (nitric oxide synthase) and CRF-(corticotropin-releasing factor) expressing neurons have been detected in the r1DR (Merchenthaler 1984; Sakanaka et al. 1987; Commons and Valentino 2002; Fu et al. 2010).

Antecedents of segmental subdivisions of the other rostral cluster nuclei that appear at paramedian or lateral intermediate positions are less evident in the literature (apart Jensen et al. 2008); in fact, relevant data are scarce and confusing, largely because the longitudinal axis across prepontine areas tended to be interpreted conventionally as a dorsoventral dimension in cross sections (see Fig. 2o). Moreover, there is little agreement on the boundaries between individual intermediate raphe subpopulations, due to the confused view that they all lie in the caudal ‘pedunculopontine midbrain’. Most authors assign all radially intermediate serotonergic populations lying in the neighborhood of the interpeduncular nucleus and the pons to the MnR (central superior) nuclear complex (e.g., Dahlström and Fuxe 1964; Törk 1990; Harding et al. 2004). In contrast, our map suggests that the serotonergic populations present in this intermediate paramedian region belong to four separate nuclear groups: CLi (a sparse population), MnR, PPnR and r3PnR, which belong to Is, r1, r2 and r3, respectively. Some earlier morphological and neurochemical data on these neurons is consistent with such subdivisions, including differences of their respective dendritic morphology and spatial distribution (see also Hale and Lowry, 2011). Dendrites parallel to the midline are typical in CLi (isthmus), whereas multipolar dendritic arbors were found in what we identify as MnR (r1) and PPnR (r2), and plexiform dendritic arrangements characterize the r3PnR cell population (Törk and Hornung 1990; Harding et al. 2004). A particular substance P receptor profile is found across these rostral intermediate raphe populations: the neurokinin receptor 1 (nkr1) is expressed selectively in CLi (Is) and r3PnR, whereas the neurokinin receptor 3 (nkr3) appears selectively in MnR (r1), and both of them are present at the PPnR in r2 (Léger et al. 2002).

The laterally migrated populations of the rostral raphe cluster (CLiW, r1–r3SuL, plus some r1DRW elements), which we found are distributed across Is, r1, r2 and r3, were described conventionally as dispersed serotonin neurons belonging to the ‘reticular formation’ (e.g., Vertes and Crane 1997; Hornung 2003); alternatively, they were lumped under the concept of ‘supralemniscal nucleus’ (Törk 1990; Jacobs and Azmitia 1992; Vertes and Crane 1997), or the ‘B9 group’ (Dahlström and Fuxe 1964). Works using immunohistochemical mapping, rather than the fluorescence methods, have emphasized the importance of these lateral serotonergic populations in rodents, in terms of the number of neurons and the longitudinal extent of their distribution (e.g., Vertes and Crane 1997). We propose that these raphe populations probably separate, respectively, from individual paramedian raphe formations in a plurineuromeric pattern: the CLiW emerges from the paramedian CLi (isthmus), the r1SuL, across both parts of r1, separates from the MnR (r1r, r1c), the r2SuL arises from the PPnR (r2) and the r3SuL spreads out from the r3PnR. We could not identify an equivalent lateral population in r4 (i.e., lateral to r4PnR).

A nucleus comparable to the CLiW is described by Puelles et al. (2007) in the chick—the so-called ‘CLi alar process’ (CLiA)—but such a concept is not found elsewhere in the literature on mammalian raphe cells. Instead, some authors assigned two lateral populations to a raphe nucleus called *pontis oralis* (PnO) (Jacobs et al. 1984; Azmitia and Gannon 1986; Hornung and Fritschy 1988; Törk and Hornung 1990). The rostral component of this PnO is located at the same place than our CLiW (isthmus); whereas the caudal PnO component seems to correspond to our r1SuL. According to our present rationale, none of these PnO entities is properly pontine, and, therefore, this name is misleading. This accounts for our proposal of new descriptive names (CLiW, r1SuL). We support adding the CLiW serotonergic population as a new nucleus belonging to the Is, distinct from the CLi.

Another aspect to discuss with regard to the ventrolateral SuL serotonergic nuclei refers to the reasons of their lateral situation relative to the medial, paramedian, or ‘authentic’ raphe nuclei. A tentative explanation of their position was offered by Steinbusch and Nieuwenhuys (1983), saying they contain “… neurons that during ontogenesis did not complete their migration toward the raphe region”. This implies the hypothesis that raphe neurons normally migrate into a paramedian position out of a more lateral (dorsal) origin, as was apparently first speculated by Harkmark (1954). Interestingly, Swanson (1992, 1993) represented all raphe nuclei in his flat brain map within a ventral part of the alar plate, indicating in the legend that some brainstem formations are mapped according to their developmental origin, rather than their adult position. Incidentally, these flat maps assign the CLi, DR and CS (or MnR) raphe nuclei, jointly with a handful of other prepontine elements, to the midbrain. The evidence supporting the mapped alar origin of raphe nuclei was not identified expressly, though perhaps fate-mapping observations of Tan and LeDouarin (1991) were considered relevant; these authors found a few labeled raphe cells after quail-chick homotopic grafting of dorsal (alar) parts of the hindbrain (note that in those studies the neurotransmitter phenotype of such ‘raphe’ cells was not determined). Apart serotonergic neurons, up to 10 different sorts of neurotransmitter-identified neuronal cell types have been found in variable numbers within the classic raphe nuclei (Nieuwenhuys 1985; Hale and Lowry 2011). It is certainly possible that some alar derivatives of r1, in particular, approach by tangential migration the raphe neighborhood, but without representing a serotonergic population; this r1 migration was studied by Lorente-Cánovas et al. (2012), and they specifically excluded the serotonergic phenotype among the migrated derivatives.

In any case, now we know that, with exception of the DR complex and some medullary paramedian elements (Jensen et al. 2008), *Nkx2.2* gene function is necessary for the development of the serotonergic phenotype in a rhombencephalic progenitor context; this condition only obtains in the paramedian basal plate adjacent to the hindbrain floor plate, due to the dependence of *Nkx2.2* induction from the floor plate source of Shh morphogen (Briscoe et al. 1999). From this point of view, we can safely assume that any laterally placed serotonergic neurons probably originated in the standard paramedian basal locus, and must have migrated afterwards to a more lateral deep, intermediate or superficial radial position within the basal plate, thus separating actively from the midline. This differential behavior suggests peculiar adhesive properties of these cells, which must be lacking in the cognates that remain at paramedian loci. We will mention below some molecular differences apparent between medial and lateral raphe populations.

A subdivision of the MnR and r1SuL serotonergic populations—both in r1—into rostral and caudal subnuclei can be envisioned. This extra-large rhombomere uniquely has differential morphological and molecular characteristics in its rostral and caudal portions (e.g., *Otx2* is expressed differentially only at the caudal r1), and several of the respective neuronal populations are somehow different (Lorente-Cánovas et al. 2012). Note for instance the clear restriction of the DTg/VTg and PDTg periventricular nuclei to r1r and r1c, respectively (Fig. 15). Vaage (1969, 1973) already proposed that the r1 domain actually should be subdivided into two neuromeres, similar to our present r1r, r1c (review in Aroca and Puelles 2005), though this idea has failed to receive general support so far, roughly for the same reason that some authors abstain from separating the isthmus from ‘r1’ (the cryptic nature of the proposed boundaries). Irrespective of how we classify it, the bipartite pattern of r1 clearly affects also the relevant raphe nuclei (present data) and neighboring tegmental nuclei, similarly as the underlying interpeduncular nucleus (Lorente-Cánovas et al. 2012). For instance, we noted that expression of *En1* is distinctly stronger at the rostral part of MnR and r1SuL than at their caudal part; *Pet1* is also stronger in MnRr than MnRc at E14.5 and E18.5, and *Lmx1b* shows a retarded upregulation at the MnRr (where it is selectively absent at E12.5), compared with MnRc.

#### The caudal cluster

The segmental organization of the raphe groups developing out of the caudal cluster—RMg, r5/r6PPy, SGeR, ROb, RPa, medullary PPy—is more controversial, due to ambiguities in the conventional anatomic description of the individual formations. The RMg initially was called ‘central inferior raphe nucleus’ by Olszewski and Baxter (1954), presumably by comparison with the ‘central superior nucleus’, or MnR (note here objectionable use of ‘superior–inferior’ terms, referring to the rostral-caudal axial dimension). Both Lidov and Molliver (1982) and Törk and Hornung (1990) placed the RMg approximately ‘between the caudal quarter of the pons and the rostral end of the inferior olive’ (which would translate into r4–r7, in our terms). Developmentally, the pons proper (pontine nuclei) ends at the caudal limit of r4; the r5 domain is well characterized by containing the trapezoid body, the superior olivary/periolivary complex and the abducens nucleus, whereas r6 selectively receives the migrated facial motor nucleus. The retrofacial part of the ambiguus motor nucleus characterizes the r7 region. The inferior olive ends rostrally somewhere in r8 and is fully absent in r7 (Marín and Puelles 1995). We interpreted that the territory referred to in the cited RMg description probably corresponded actually to r5–r7, since r4 shows very few raphe cells (see below), and we know that the pons used to be extended conventionally at least into the area we identify as r5. The rostralmost RMg cells (accurately labeled experimentally as r5-derived by Jensen et al. 2008) are otherwise described as coinciding with the trapezoid body (Hornung and Fritschy 1988), at section levels through the facial genu and the abducens nucleus (Törk and Hornung 1990), or the superior olive (Jacobs and Azmitia 1992), all of which are r5 anatomic markers. In our material, the RMg population seems to be larger and more compact within r6 (because of less decussating fibers?). In fact, the RMg topography proposed by Steinbusch and Nieuwenhuys (1983) restricts it completely to r6, since these authors hold that it is coextensive with the facial motor nucleus. We agree with these authors that the raphe cells found in r7, intercalated between the facial nucleus and the superior olive, are best assigned to the RPa/ROb complex. We conclude that this classic nucleus in any case occupies a retropontine position within the pontomedullary region, and we tentatively define its rhombomeric extent as occupying r5 and r6.

Similar calculations were done for placing the other caudal raphe formations, since available descriptions were rather variable. The rostral end of the ROb was described by Jacobs and Azmitia (1992) as at level with the VI nucleus or VI nerve root (r5), while the RPa was reported by other authors (Olszewski and Baxter 1954; Taber et al. 1960) to stop rostrally at the level of the middle of the motor facial nucleus (r6) or, alternatively, at the rostral pole of the inferior olive (r8; Hornung and Fritschy 1988; Jacobs and Azmitia 1992). These differences in description probably obey to variations in the sectioning plane. We think that it is not evident from the literature that ROb and RPa coincide in reasonable cross sections with RMg, though they do coincide with each other. This is particularly clear when sagittal sections are studied (Fig. 3). We therefore suggest that ROb and RPa both begin rostrally in r7, once RMg ends in r6. The r6/r7 boundary happens to correlate with the change from overt rhombomeres to cryptorhombomeres (Cambronero and Puelles 2000; Watson et al. 2010), as well as with the transition of the hindbrain pontine and retropontine molecular domains controlled by the *Hox1*–*Hox3* gene paralogs into the domains controlled by the *Hox4*–*Hox7* paralogs (Marín et al. 2008); the r6/r7 boundary therefore may explain the RMg versus ROb/RPa structural and typological transition. The ROb and RPa complexes emerge accordingly as being coextensive with the cryptorhombomeres r7–r11. Since these developmental units tend to develop very similar structures (metamery, or plurineuromeric regularity), causing the appearance of apparently continuous columnar plurineuromeric complexes, this would explain that both ROb and RPa have been always interpreted as single entities. An increase in shared morphological characteristics is noted throughout in these caudal hindbrain developmental units (predominant columnar structure of all nuclei in the caudal medulla).

Apart of the r5 and r6 portions, we propose a dorsoventral subdivision of the RMg complex into a slightly dispersed intermediate stratum component (RMgD), and a more compact ventral or superficial part (RMgV); this notion was already introduced by Puelles et al. (2007) in the chick atlas. There is evidence that the RMgD and RMgV cell populations have different patterns of differentiation, so that RMgV develops first (Lidov and Molliver 1982; Wallace and Lauder 1983). Interestingly, there is also some evidence of neuronal typological differences within the retropontine RMg complex, which may correlate with the alternative r5 versus r6 topographies (see Hornung and Fritschy 1988). As regards chemoarchitectonic properties, whereas SP neurons are detected in both r5 and r6 parts of RMgD, TH neurons are detected at the r5RMgV, but are absent at r6RMgV (Allen Developing Mouse Brain Atlas; http://www.developingmouse.brain-maps.org; Halliday et al. 1988; Rikard-Bell et al. 1990; Poulat et al. 1992; Wu et al. 1993; Hornung 2003). In any case, TH neurons probably are migrated from non-raphe sources, and would not be very significant.

Differential segmental patterns are therefore less obvious in the cryptorhombomeric raphe nuclei (ROb, RPa; Steinbusch and Nieuwenhuys 1983; Hornung and Fritschy 1988; Jacobs and Azmitia 1992; Bjarkam et al. 1997; Cambronero and Puelles 2000; Nieuwenhuys et al. 2008). They belong to a hindbrain subregion that is devoid of overt rhombomeric limits, and adjacent developmental units tend to be homogeneous in their histogenesis, irrespective of the local differential patterns of expression of *Hox* gene paralog groups (see Marín et al. 2008). Raphe nuclei of r7 and r8 seem to lack cells expressing SP or TH, unlike raphe nuclei in r5 and r6; such cells reappear in the r9 and r10 ROb units (Del Fiacco et al. 1984; Halliday et al. 1988; Rikard-Bell et al. 1990).

Lateral or parapyramidal serotonergic cells of the caudal cluster appear early in development, as observed at the rostral cluster. They extend longitudinally between r5 and r11 (Lidov and Molliver 1982; Wallace and Lauder 1983; Aitken and Törk 1988; present results). The serotonergic parapyramidal cells within r5 and r6 are conventionally described as located in the ‘lateral paragigantocellular nucleus’ (LPGi) (Jacobs and Azmitia 1992), or in the ‘rostral ventrolateral medulla’ (Törk 1990; Harding et al. 2004). Their lateral position results from a migration pattern that resembles the one postulated for the rostral SuL cluster (Hawthorne et al. 2010), but associated in this case to the source of the RMg cells in r5 and r6. We feel it may be clarifying to refer to these cells as r5 and r6 parts of the PPy column, to emphasize the regional similarity with the more caudal medullary PPy group.

The medullary PPy was identified within r7–r11, surrounding laterally the inferior olive (once the inferior olive ends, these cells surround the pyramidal tracts, or intermix with the pyramidal decussation). These cells were previously vaguely included in the RPa nuclear complex, or their position was defined as occupying the ‘caudal ventrolateral medulla’ (Törk 1990; Harding et al. 2004). We thus propose to classify them as a continuous series of rhombomeric PPy raphe cell groups (r7PPy–r11PPy).

A peculiarity of the caudal cluster is the general absence of periventricular serotonergic populations. This pattern is permanent in the cryptorhombomeres r7–r11, but some small 5-HT-immunoreactive periventricular neurons appeared in our postnatal material, associated to the supragenual area in the pontomedullary rhombomeres r5 and r6. They correspond to the ‘extraraphe’ cells described by Olszewski and Baxter (1954), or the B4 cell group mentioned by Dahlström and Fuxe (1964). A retarded postnatal neurogenesis and differentiation of these paramedian cells does not seem plausible, since hindbrain neurogenesis is held to stop long before; late birthdates might be compatible with a source in the r5–r6 rhombic lip. It will be necessary to investigate a possible tangential migration of these cells into this position; the possibility that these cells are non-neuronal has to be investigated as well, since we did not detect any expression of *Pet1* at this place at either embryonic or postnatal stages. Indeed, some hypothalamic tanycytes were found to be immunoreactive for serotonin (Steinbusch and Nieuwenhuys 1983) due to transmembrane transport of monoamines from the surrounding medium (Ugrumov et al. 1989; Ugrumov 1997; Hansson et al. 1998).

### The raphe pontis nucleus in r4

Our results demonstrate the presence of serotonergic neurons in r4 in mouse brains at embryonic and postnatal stages (our r4PnR cell group). However, the absence of serotonergic neurons in r4 is an accepted dogma in the field (e.g., Jensen et al. 2008). Studies focused on the molecular profile of this rhombomere at early stages demonstrated a sustained production of branchiomotor neurons (bMNs) and postulated, as a consequence, a local inhibition of serotonergic neuron production (Pattyn et al. 2003; Jacob et al. 2007). Such inhibition is attributed to collateral effects of the transcription factor *Phox2b*, maintained locally by *Hoxb1*, on the production of bMNs (Pattyn et al. 2003). These studies were restricted to relatively early stages (E9.5–E11.5), when bMNs of the facial motor nucleus are generated (Goddard et al. 1996; Studer et al. 1996). Differentiation of serotonergic neurons apparently was not explored at later stages, which is when we detected such neurons in r4 (from E12.5 onwards). Thus, our results indicate instead a heterochronic biphasic pattern in r4, first with prolonged local generation of bMNs, followed by delayed production of a few serotonergic neurons, possibly bespeaking of a skewed probabilistic control mechanism of the fate choice done by the relevant postmitotic neurons.

We observed that r4 pontine raphe neurons are not transient, since they persist at postnatal stages (present results) and in adults (data not shown). Also, they are not a peculiarity of the mouse brain, since it is possible to confirm their existence in other mammals, though the r4RPn nucleus tends to be classified as a rostral component of RMg (Taber et al. 1960; Skagerberg and Björklund 1985; Hornung and Fritschy 1988). Similar pontine r4 raphe cells were found as well in sauropsides (chick; Cambronero 1999; reptiles; Rodrigues et al. 2008; their Fig. 1).

In addition to a delayed and diminished production of serotonergic cells, there exists also a peculiar transcriptional regulation of the serotonergic phenotype in r4, at least in the mouse. The r4PnR neurons are positive for *Gata3*, *Lmx1b* and *Pet1*, but we did not detect the expression of *Gata2*, *Tph2* and *Slc6a4* in this group. The absence of *Gata2* signal suggests that this gene is not necessary to determine the serotonergic phenotype in r4, this role probably being assumed solely by *Gata3*, similarly as occurs in the caudal cluster (van Doorninck et al. 1999; see below). The most striking molecular deviation of this serotonergic group is the very low or absent signal of *Tph2*, despite the normal expression of *Pet1* and *Lmx1b*, and the presence of immunoreactive 5HT. A plausible interpretation predicts the existence of a particular isoform of tryptophan hydroxylase (TPH) in r4, which is not detected by our *Tph2* riboprobe. An alternative explanation is that these neurons are unable to synthesize TPH enzymes, due to the absence of some transcription factor necessary to regulate the expression of its messenger (for example Gata2), but they have the capacity to synthesize the serotonin transporter (Slc6a4)—under independent regulation by *Pet1* and *Lmx1b*—and, therefore, can take up serotonin present in their environment. Although we did not detect ourselves *Slc6a4* expression during mouse development (negative data attributed to malfunctioning of the probe used), positive data of its expression at E15.5-P14 are found in the Allen Developing Mouse Brain Atlas (http://www.developingmouse.brain-map.org), supporting the latter hypothesis. Neurons that take up serotonin, but do not synthesize it, are known in the hypothalamus (Ugrumov et al. 1989; Ugrumov 1997; Hansson et al. 1998). The status of these r4RPn neurons as *bona fide* serotonergic neurons (Hoffman et al. 1998) is therefore still controversial, until the doubt about TPH is resolved.

### Serotonergic populations outside the hindbrain

We detected only two serotonergic populations lying outside the rhombomeric territory. These were placed, respectively, at the rostral and caudal ends of the hindbrain raphe system: the midbrain mDR cell group and a small serotonergic group in the cervical spinal cord. Immunoreactive serotonergic neurons start to appear in the caudal midbrain at E14.5 (though some *Pet1*-expressing cells were found at E12.5), and form the sizeable mDR group just rostral to the isDR. The mDR, which corresponds to what some authors identify as ‘rostral DR nucleus’ (Hale and Lowry 2011), is restricted to the small preisthmic region (recently redefined as mesomere 2—m2—of the midbrain; Hidalgo-Sánchez et al. 2005; Martínez et al. 2012; Puelles et al. 2012b; Allen Developing Mouse Brain Atlas, http://www.developingmouse.brain-map.org). In contrast to conventional attribution of the rostral raphe cluster as a whole to either the midbrain, or the hindbrain (Jensen et al. 2008), these are serotonergic neurons that are truly present in the adult midbrain, as long as the caudal end of the *Otx2* expression domain is accepted as the midbrain–hindbrain boundary (Puelles et al. 2012b). The genetic profile of the mDR resembles that of its neighbor, the isDR, suggesting that these neurons may migrate tangentially from the isthmus, where they would be born. Alternatively, the parallel characteristics may be due to similar effects produced by the isthmic organizer on both m2 and isthmic progenitors. The migration hypothesis is supported by their early absence at the midbrain at E12.5, and their gradual appearance at E14.5, shaped as a rostrally pointing spike connected with the isDR across the molecular *Otx2*-labeled MHB. This boundary is known to be permissive to tangential neuronal migration between midbrain and hindbrain in both senses. Kala et al. (2008) studied in transgenic mice the distribution of MHB-Cre labeled neurons derived from rostral ‘r1’ (meaning essentially the isthmic region), and found evidence of labeled cells entering the caudal midbrain (what we interpret as m2), presumably representing or including the mDR cells. Similarly, Zervas et al. (2004) studied the *Wnt1*-related lineage (*Wnt1*-CreER), supposedly restricted to the midbrain, finding that the labeled cells are majoritarily dopaminergic, but that they intercalate with some unlabeled serotonergic neurons (their Fig. 3c); these would have migrated from the isthmus; however, they also found some isolated double-labeled cells. Puelles et al. (2004) suppressed *Otx2* expression in the basal plate of the midbrain, implicitly modifying the local molecular identity, or the site of the functional MHB. This caused an expansion of *Nkx2.2* expression in the midbrain basal plate, and a consequent reduction of dopaminergic neurons in favor of serotonergic ones (the authors did not determine whether the latter were generated locally, or migrated from the isthmus). Curiously, we observed that as the mDR starts to form, *Otx2* diminishes or disappears at its location (see our Fig. 4d–f). It is unclear whether down-regulation of this gene precedes (and maybe causes) the appearance/migration of the mDR, or is rather a consequence of its migratory formation. In any case, the phenomenon bespeaks of a possible cross-repressive molecular interaction between m2 and isthmic derivatives. On the other hand, apart of isthmic serotonergic neurons that seem to invade the midbrain, there exist also midbrain dopaminergic neurons that seem to invade secondarily the isthmic tegmentum (LP, unpublished observations). Finally, it is interesting to note that the observations of Sako et al. (1986) and Cambronero (1999) on DR serotonergic neurons in the chick did not disclose any significant mDR homolog, since only few isolated elements appeared transiently rostral to the MHB.

Similarly as other authors (e.g., Törk and Hornung 1990; Jacobs and Azmitia 1992), we found 5HT-positive neurons in the upper cervical levels of the spinal cord. These neurons appear approximately at the same time as their rostral neighbors in the RPa and ROb nuclei (at E12.5, according to our results). These data suggest an in situ origin, rather than a migration, though we cannot discard that possibility. Their low number might be an effect of repressing retinoic acid signals from the caudal secondary organizer (Díez del Corral and Storey 2004).

### Segmental organization of raphe nuclei correlated with their developmental genoarchitecture

The raphe nuclei are distributed rostrocaudally throughout the rhombencephalic paramedian basal plate, with minimal invasion of midbrain and spinal cord. This represents accordingly a shared histogenetic feature of the whole set of rhombomeres constituting the hindbrain tagma (isthmus, r1–r11). This feature probably can be attributed to a comparable influence of notochordal and floorplate-derived Shh signals on this part of the neural tube, with immediate effects on both the serotonergic and motoneuronal populations (Briscoe et al. 1999). Further effects downstream of Shh, involving *Nkx2.2*, *Lmx1b* and the *Gata 2/3* genes in the absence of *Otx2* apparently lead to the serotonin transmitter phenotype, with associated differentiation markers. Notwithstanding this common causal scenario repeated along the hindbrain AP axis, there are overlapping, variously nested expression patterns of *Hox* homeodomain genes and other hindbrain differential molecular determinants, which correlate causally with hindbrain segmentation (Lumsden and Krumlauf 1996; Marín et al. 2008). The resulting differential genoarchitectonic profiles of the rhombomeres provide regional differences in genomic regulation, which allow in principle individual raphe nuclei to become distinct one from another, as occurs with other derivatives of these developmental units (motor and sensory populations, reticular cells, etc.). Our present approach included examining whether given molecular features differ between the diverse raphe populations, presumably as a result of their segmental identity and particular histogenetic conditions (e.g., radial or lateral migration).

**Fig. 13:**
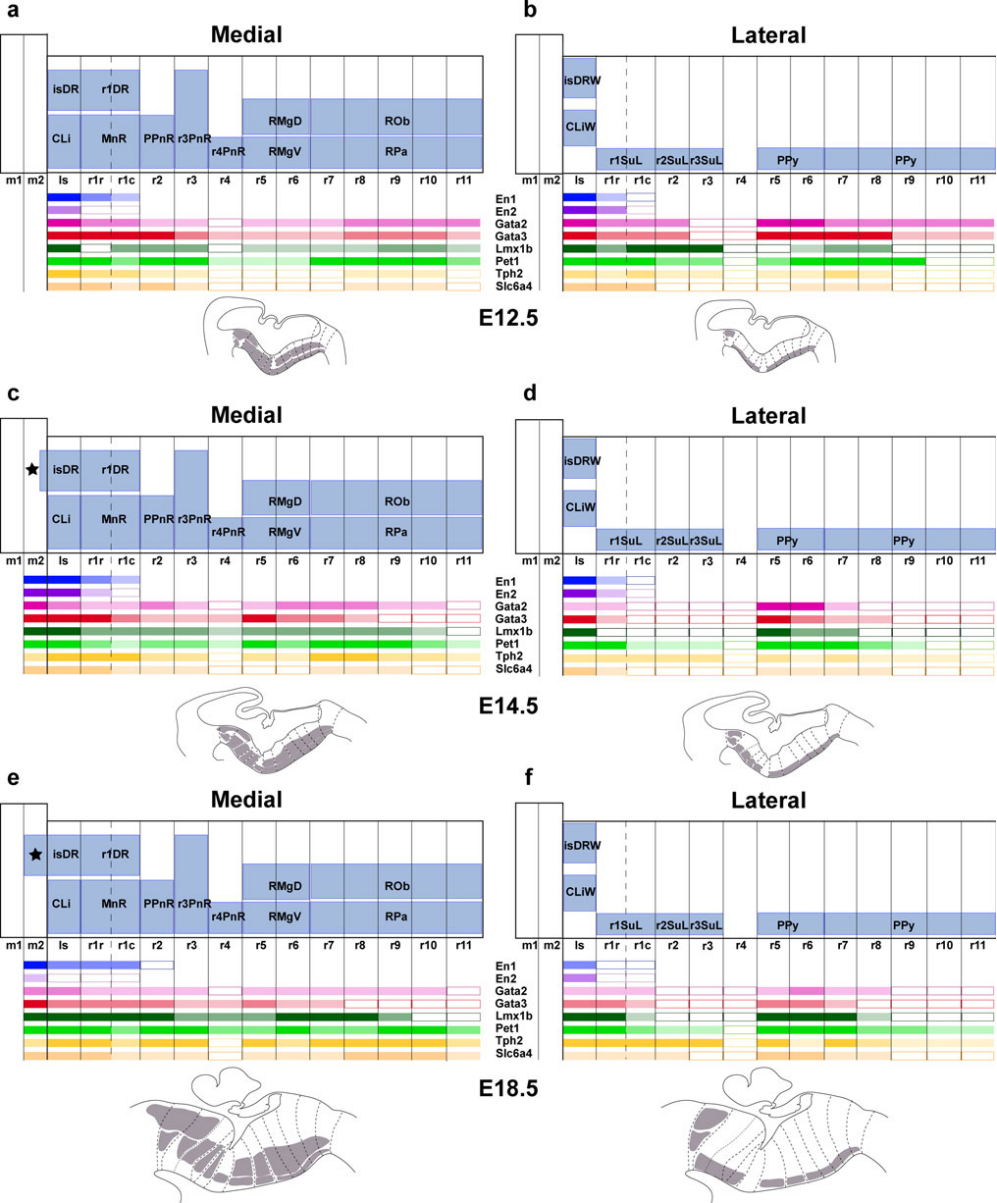
Schematic synthesis of the studied molecular profile of the mouse raphe nuclei during embryonic development. Medial and lateral raphe nuclei are represented at three different embryonic stages, with a corresponding pair of schemata of results obtained either next to the midline or more laterally: E12.5 (**a**, **b**), E14.5 (**c**, **d**), E18.5 (**e**, **f**). Below each *rectangular *schema of the segmented hindbrain, with mapped raphe populations filled-in in gray, the respective intensity of gene expression for eight *color-coded* markers is represented by *different color hue*. Underneath is added a realistic schema of the corresponding sagittal section thus mapped. *Black asterisks* in **c** and **e** represent the expanding mDR nucleus

Segmental identity in terms of a specific set of active genes is imprinted early on the neuroepithelial progenitor cells, generally shortly before neurogenesis begins, and such identity may be inherited or diversified subsequently in particular neuronal derivatives, as emergent *genoarchitecture* (Ferrán et al. 2009). Serotonergic neurons share common progenitors with visceromotor (vMN) and branchiomotor (bMN) neurons (Briscoe et al. 1999; Pattyn et al. 2003; Jacob et al. 2007), though there are exceptions; there are no vMNs and bMNs at the Is and r1, and r4 is supposed to lack serotonergic neurons, or produces few of them (Weilan et al. 1998; Briscoe et al. 1999; Ding et al. 2003; Pattyn et al. 2003; Jacob et al. 2007). Didactic generalization to the whole hindbrain of results obtained in individual rhombomeres can omit segmental particularities worthy of consideration. In the end, developmental programs must exist in each rhombomere that enable production of specific visceromotor and/or branchiomotor neurons, plus specific serotonergic neurons, among other specific anatomical derivatives. Our results on genes expressed in postmitotic serotonergic neurons show that there exist indeed peculiarities related to rhombomeric topography, as well as some variations occurring during development (Fig. 13; see also Wylie et al. 2010).

Among the genes examined, *En1* and *En2* are expressed at the midbrain and rostral hindbrain, down to r1, and thus their signaling has a restricted position with regard to the whole set of raphe nuclei (Wylie et al. 2010; Fox and Deneris 2012; present results). Data from *En* mutants (Simon et al. 2005) indicate there is no phenotype caudal to r1. The DR, CLi and MnR nuclei are lost, but not so PPnR (in r2) and r3PnR (for clarity, we interpret the reported data according to our terminology). In our material, *En2* signal is first restricted (up to E16.5), in a gradient decreasing caudalwards, to the ventricular zone of Is-r1r, but is lost by E18.5, suggesting a transient role in the differential specification of the local serotonergic derivatives (isDR, r1DR, CLi and MnR). It is tempting to speculate that the *En2*-positive domain, representing the range of Fgf8 morphogen signaling from the isthmic organizer, may embody a local molecular context that prohibits local differentiation of bMNs (the rostralmost hindbrain bMNs pertain to r2), and only allows MNs at a restricted rostral locus in the isthmus (trochlear nucleus). The domain of *En1* expression essentially overlaps spatially and in its gradiental aspect that of *En2*, but expression extends also to the periventricular and intermediate strata of serotonergic cells in the mantle. This pattern persists at least until postnatal stages, suggesting a supportive role in the maintenance of some aspect of the local serotonergic cells (personal observations, Allen Developing Mouse Brain Atlas, http://developingmouse.brain-map.org/). A minor variant, which can be attributed to migratory displacement, is the presence of a patch of *En1*-positive neurons in the PPnR (in r2; Jensen et al., 2008). It has been postulated that *En1* is involved in the maintenance of the serotonergic phenotype in the hindbrain, similar to its role with regard to the dopaminergic phenotype in the midbrain (Simon et al. 2005). This phenotypic duality seems related to differential fate regulation due to overlapping expression of *Otx2* in the midbrain (Brodski et al. 2003; Puelles et al. 2004; Simeone et al. 2011).

The other six genes studied by us (*Gata2*, *Gata3*, *Lmx1b*, *Pet1*, *Tph2* and *Slc6a4*) are expressed in most serotonergic groups, implying a fundamental relationship with the neurotransmitter phenotype (Deneris and Wyler 2012), rather than with differential segmental identity. Expression of *Gata2* precedes that of *Gata3* (Nardelli et al. 1999). As happens in hematopoietic cells (Ferreira et al. 2007), variations in the regional expression levels of the *Gata* genes may relate to different dosage requirements in distinct serotonergic nuclei (we observed highest levels of transcription in the lateral raphe nuclei of r5 and r6). Interestingly, the functional roles of these two genes differ as regards the rostral and caudal raphe clusters, since the former selectively requires *Gata2,* whereas the latter, and specially the ROb nuclear complex, needs *Gata3* (Nardelli et al. 1999; van Doorninck et al. 1999; Craven et al. 2004). These data reveal a particular genetic regulation requirement for the cryptorhombomeric serotonergic groups, or at least for some particular raphe populations within them. In any case, the onset of *Gata3* expression correlates with that of *Lmx1b* and *Pet1*.

*Lmx1b* and *Pet1* appear to play important roles in the differentiation and maintenance of the serotonin phenotype, though the ampler initial expression domain of *Lmx1b* in the hindbrain—notably in the periventricular stratum throughout the length of the hindbrain—suggests that it controls as well other neuronal phenotypes. As development advances, these non-serotonergic extra populations gradually lose the *Lmx1b* signal. Note that *Lmx1b* is expressed likewise initially along the midbrain, diencephalic and hypothalamic basal and floor plates, in connection with the production of dopaminergic neurons and probably also other local cell types. *Lmx1b* apparently acts upstream of *Pet1* in hindbrain serotonergic neurons (Hendricks et al. 1999; Pfaar et al. 2002). Although the loss-of-function phenotypes of both genes are similar (Hendricks et al. 2003; Ding et al. 2003), we observed differences in their respective hindbrain expression patterns. In the rostral cluster, *Lmx1b* signal appears in a rostro-caudal gradient (Ding et al. 2003; present results), not observed with *Pet1*; this gradient is consistent with the *En1/En2* gradients and the spatial gradient of differentiation of 5HT-immunoreactive neurons. This pattern is maintained into adulthood, probably due to a maintenance function of the serotonergic lineage similar to that observed for the dopaminergic lineage (Smidt et al. 2000), which would seem to require highest dosage rostrally. At E14.5 and E18.5 we observed distinctly stronger expression of *Lmx1b* at the MnRr than at MnRc.

In the caudal cluster, *Lmx1b* signal has a rostrocaudal gradiental expression pattern which roughly agrees again with the spatial pattern of maturation of 5HT neurons; these differentiate first in r6 (normally the paired rhombomeres are advanced in neurogenesis relative to unpaired ones), extending immediately afterwards to r5 (Cambronero 1999) and gradually into the r7–r11 series (Wallace and Lauder 1983; Pattyn et al. 2003). The medullary groups may have a lower requirement of *Lmx1b* for their differentiation, if other transcription factors, such as *Gata3* or *Pet1* play a complementary role. We observed that the *Lmx1b* signal is patchy and is completely switched off at the caudal cryptorhombomeres at late developmental stages; this suggests that this gene does not have a relevant function in serotonergic lineage maintenance in the medulla. *Pet1* might replace *Lmx1b* in this regard, since its expression is more homogeneous, and, moreover, it is known to be required for the maintenance of the serotonergic phenotype in adults (Liu et al. 2010; Song et al. 2011).

At early stages, *Pet1* signal presents some peculiarities relative to segmental raphe populations, since it first appears in a non-gradiental, irregular pattern (Fig. 13). These differences apparently reflect heterochronic rhombomere-specific regulatory programs for the expression of this gene. Consistently with this idea, the *Pet1*-null mutant conserves selectively *Pet1*-positive populations at the isthmus, probably due to the agency of a separate enhancer (Hendricks et al. 2003; their Fig. 2). This serotonergic population roughly coincides with the *Tph2* positive neurons that are conserved in *Tph2*-conditional mutants (Kriegebaum et al. 2010), suggesting a close relationship in the regulation of *Tph2* expression by *Pet1* at isthmic levels. On the other hand, *Pet1* and *Lmx1b* are jointly implicated in the regulation of *Slc6a4* throughout the set of raphe primordia (Hendricks et al. 1999, 2003; Zhao et al. 2006), but the expression of the latter is delayed compared with that of *Tph2*. This marker also shows various heterochronic aspects among specific segmental serotonergic groups (Fig. 13).

**Fig. 14:**
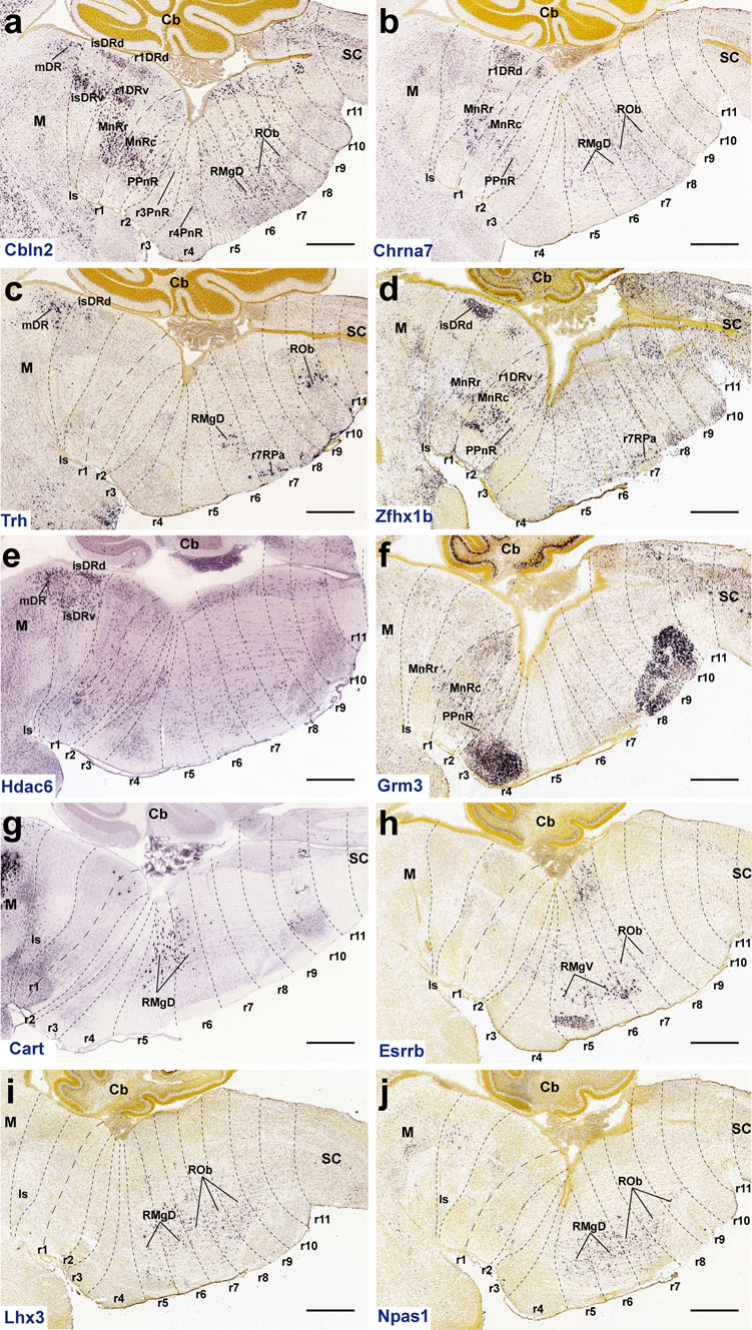
Selected results of a search done in the Allen Adult and Developing Mouse Brain Atlases, looking for mouse genes with restricted expression patterns within the raphe nuclei (irrespective of other domains of expression). The gene tag is indicated at the *lower left* corner of each panel. **a–d** Genes with expression restricted to some specific raphe subgroups of the rostral and caudal clusters. **e**, **f** Genes with expression restricted to only some nuclei of the rostral cluster. **g–j** Genes with expression restricted to only some nuclei of the caudal cluster. The stages are P4 (**d, f, h, i**, **j**), P14 (**a–c**) or P56 (**e**, **g**). *Scale bar* 500 μm

We searched for genes displaying an expression pattern restricted to some raphe nuclei in the Allen Mouse Brain Atlas database (http://mouse.brain-map.org). We found genes expressed in some cell aggregates or scattered cells in both clusters, and other genes expressed only in some rostral or caudal cluster subdivisions. We noted that such genes are not limited to those involved in the specification or maintenance of the serotonergic phenotype. Some of the genes identified—*Cbln2*, *Grm3, Chrna7*, and *Trh*—are probably involved in the modulation of serotonergic functions. *Cbln2* is implicated in the formation of a kind of excitatory synapse, and in synaptic communication, in the central nervous system (Eiberger and Schilling 2012). In serotonergic subpopulations, it is possibly related to glutamatergic modulation of this neuronal phenotype (Soiza-Reilly and Commons 2011), as occurs likewise with *Grm3* (Harrison et al. 2008). The function of *Chrna7* is less clear, but the serotonergic and cholinergic systems apparently modulate themselves mutually in some cognitive functions such as learning and memory (Garcia-Alloza et al. 2006); the regionally restricted expression of this gene within the raphe system suggests that such modulation may be particularly relevant in the serotonergic subpopulations where it is expressed. *Trh* is a hormone implicated in the modulation of arousal, cognition, motor functions and pain (Boschi et al. 1983; Nillni and Sevarino 1999), in addition to its endocrine actions. Curiously, *Trh* is expressed selectively at the RMgV (the ‘rostral ventromedial medulla’ of Porreca et al. 2002; see our Fig. 14c), which is involved in modulation of ascending pain signal transmission, thanks to its descending projections to the dorsal horn of the spinal cord.

Other genes with restricted expression within the raphe populations remain devoid of a clear role where they appear expressed. *Cart* induces neurite elongation and ramification in dopaminergic, hippocampal, retinal and motoneurons primary cellular cultures (Rodrigues et al. 2011); maybe it is related with some local aspects of pre- and postnatal development of serotonergic subpopulations, including synaptogenesis. *Hdac6* reportedly has a role in glucocorticoid-receptor-related homeostasis of particular raphe circuits related to social behavior, particularly in the DR nucleus (Espallergues et al. 2012). No known function can be attributed to the transcription factors *Zfhx1b* and *Lhx3*, and the orphan nuclear receptor *Esrrb,* in the serotonergic subpopulations and neighboring cells in which they are expressed.

### Do segmental components of the raphe nuclei have heterogeneous connectivity patterns?

The diverse connectivity patterns so far described for the raphe nuclei probably are best explained by their segmental organization. A recent publication of Bang et al. (2012) indeed strongly supports that idea, even though the flatmap graphic representation of their results in their Fig. 5 is surprising in lacking transverse rhombomeres at all, and inexplicably represents median or paramedian raphe or dopaminergic cell populations (VTA, MR, DR) at a considerable distance from the flatmap midline. Molliver (1987) referred to serotonin projections as evidencing “*multiple neuronal subsystems that have high degree of specificity and precision in their organization*”. The modular character of the rhombomeric derivatives allows in principle the emergence of these characteristics, since individual subpopulations may develop quite diverse sets of efferent and afferent connections, while conserving some shared features (e.g., joint axonal navigation rules with opportunistic properties). A segmental connectivity pattern is clearest at the rostral cluster nuclei, which also have received more attention in hodologic studies.

There exists some degree of segmental hodologic specialization of the isthmic DR subnuclei—isDR jointly with the mDR—versus those derived from r1. These patterns were lumped by Bang et al. (2012) because of their joint labeling of midbrain, isthmic and r1 raphe component projections derived from their composite ‘r1’ domain. The mDR/isDR neurons apparently preferentially project upon centers involved in motor control—substantia nigra, caudate/putamen—while the r1DR neurons project to limbic system formations—hippocampus, locus coeruleus—(Fuxe et al. 1977; Köhler and Steinbusch 1982; O’Hearn and Molliver 1984; Imai et al. 1986; Mamounas et al. 1991; Vertes 1991; Jacobs and Azmitia 1992; Waselus et al. 2006). Some segmental specialization of efferences apparently also occurs in other populations—MnR, PPnR, PnR, RMg, and medullary raphe nuclei—although the available data are scarce and often contradictory.

An example that illustrates this is the attribution of a variety of efferent targets to the MnR in the literature, probably due to the fact that many authors lump in this complex different sets of raphe neurons actually located in r1, r2, or even r3 (Törk and Hornung 1990; Vertes et al. 1999). According to earlier, less clearcut evidence, the MnRr apparently sends axons to the amygdala, hippocampus, septum, diagonal band and (probably) the rostral IP, while the MnRc projects specifically to the hypothalamus (some authors emphasize the suprachiasmatic nucleus), DR, ventral tegmental area, substantia nigra pars compacta, and (probably) the caudal IP nucleus (Imai et al. 1986; Vertes and Martin 1988; Meyer-Bernstein and Morin 1996; Vertes et al. 1999). Taking into consideration the relevant selective labeling of r2 raphe projections reported by Bang et al. (2012), it would seem that the cells innervating selectively the suprachiasmatic nucleus derive from r2, even if they occupy a place in MnRc, which lies in caudal r1. Such movements to neighboring raphe domains are by no means impossible (Jensen et al. 2008). Another selective projection of r2 raphe cells (it is unclear whether we deal here with PPnR) is to the posterior periventricular nucleus of the thalamus (Bang et al. 2012). It is otherwise very difficult to dissociate among all available data those projections that may concern specifically the PPnR (r2), in contrast to the MnRc. It is therefore highly plausible that some of the heterogeneous connections attributed to the MnRc actually belong to the PPnR, or to cells migrated from r2 into r1.

Few hodological studies mention specific connections attributed to the PnR (r3PnR in our interpretation), though some results suggest that group projects mainly to visuomotor centers, such as preoculomotor reticular neurons, the superior colliculus and some pretectal nuclei, in addition to the cerebellar vermis (Bobillier et al. 1976; Päällysaho et al. 1991). However, none of these targets was mentioned in the recent description of r3 + r5 raphe projections (Bang et al. 2012); these authors emphasized instead forebrain projections largely shared with ‘r1’ and r2 raphe neurons, with subtle differences (mainly at the neocortex and amygdala), and connections directed toward other raphe nuclei (MnR, DR), the lateral parabrachial nucleus, the locus coeruleus, the anterior or ventral tegmental nucleus (a target we would interpret rather as the rhabdoid nucleus, due to its characteristic size, shape and paramedian position behind the decussation of the brachium conjunctivum) and the dorsal tegmental nucleus.

The supralemniscal raphe nuclei extending across r1–r3 also show some differences in connectivity, once the data available are poured into our interpretive schema. The study of Vertes and Martin (1988) reported efferents to the central IP subnuclei, retrorubral area, substantia nigra pars reticulata, anterior pretectal nucleus, the thalamic anterior intralaminar complex, the suprachiasmatic nucleus and some other hypothalamic centers, such as the retromammillary nucleus and the mammillary body, and the preoptic area, which we believe (according to the retrograde mappings themselves) map selectively to the r1SuL, which the authors identified as the ‘nucleus pontis oralis’. In contrast, we interpret retrograde hodological mappings of reported projections to the MnR (Stratford and Wirtshafter 1988), the prethalamic reticular nucleus (Rodríguez et al. 2011), the arcuate and ventromedial hypothalamic nuclei (Willoughby and Blessing 1987) and the entorhinal cortex (Köhler and Steinbusch 1982) as labeling rather selectively the r2SuL and/or r3SuL cell populations.

In the case of the caudal raphe groups, some confusion results from the circumstance that their descending axons send collaterals to diverse spinal cord segments; the shared axonal navigational properties have probably blurred any specific segmental origins of given connectivity patterns (Harding et al. 2004). Even so, it was shown that r5RMg neurons project specifically to the dorsal horn of the spinal cord (Ruda et al. 1982; Hylden et al. 1986; Jacobs and Azmitia 1992), whereas r6RMg neurons project instead to periventricular layer X of the cervical spinal cord and to the spinal trigeminal nucleus (Beitz 1982; Azmitia and Gannon 1986; Jacobs and Azmitia 1992). There are also striking differences between the efferents of the r5 and r6 parts of the PPy formation. The r5PPy neurons project to the intermediate sensorimotor zone of the spinal cord, where the autonomic sympathetic preganglionic neurons are found (Bowker et al. 1982), while the r6PPy neurons establish connections with the brainstem ventral respiratory groups (Ellenberger and Feldman 1990; Morillo et al. 1995), the spinal dorsal and ventral horns, and the sacral parasympathetic preganglionic population (Hermann et al. 2003).

As regards the medullary raphe groups, the paramedian elements generally project to motor neurons of the brainstem (ROb; Felten and Sladek 1983) and the ventral horn of the spinal cord (ROb and RPa; Azmitia and Gannon 1986; Sasek et al. 1990; Veasey et al. 1995). In its turn, the medullary parapyramidal nuclei (r7–r11PPy) connect generally with the preganglionic neurons of the autonomic nervous system (Sasek et al. 1990). In these cases, potential rhombomeric specificities have not been explored, and do not transcend from published material.

Within each rhombomere, the radial and medio-lateral subdivisions of the local serotonergic nuclei apparently may have differential efferent targets (Imai et al. 1986; Vertes et al. 1999). The clearest data supporting such specialization belong to the isDR complex, since its medial subdivisions (isDRd, isDRv) send ascending projections to somatosensory centers—somatosensory thalamus (trigeminal) and cortex—(Kirifides et al. 2001; Lee et al. 2008), while the lateral subdivision (isDRW) projects instead to visual centers (Pasquier and Villar 1982; Villar et al. 1988; Waterhouse et al. 1993). More detailed genoarchitectonic studies of raphe subdivisions and their development are needed to examine the causes of such connective specialization.

Bang et al. (2012) have further underlined the existence of selective innervation of some serotonergic raphe cells by collaterals coming from specific rhombomeric populations (for instance, DR axons project into MnR, RMg and ROb, r2-derived serotonergic terminals surround non-r2 MnR neurons, and r3 + r5 originated raphe axons selectively reach the DR). These synapses are held to generate collateral modulatory inhibitory effects via the 5HT1A receptor.

The general conclusion from this analysis is that all connections of the raphe nuclei need to be examined further taking in consideration their rhombomeric position and boundaries. The same applies to afferences to these nuclei. Some mutant mouse lines are presently available (and more will accrue) that are useful to test the role of specific rhombomeres in the development and function of given connections. Such an effort should enhance significantly our understanding of the functions of serotonergic signaling in general, probably discriminating a number of discernible subsystems, and throwing light on various pathophysiological aspects.

## Conclusions

**Fig. 15:**
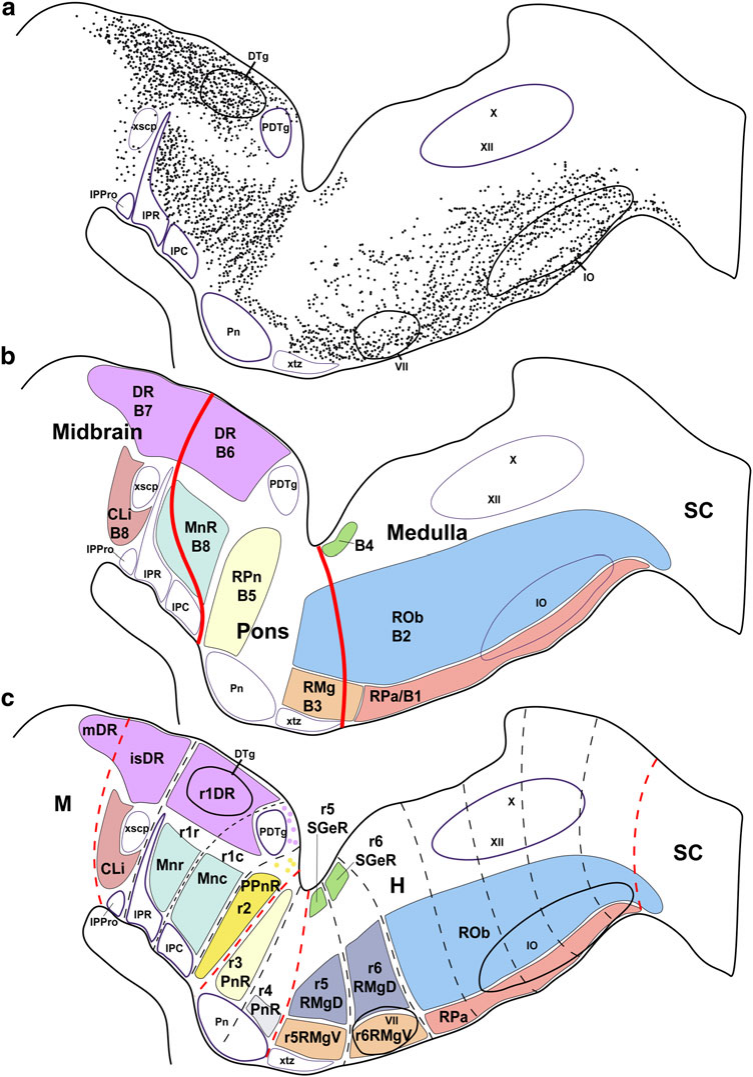
Comparison of old and new raphe classifications. **a** Schematic median projection of paramedian raphe serotonergic cells taken from a postnatal mouse specimen, showing relative cell densities. **b** Schema of the conventional identification of 9 paramedian raphe nuclei across midbrain, pontine and medullary territories, the latter delimited roughly according to the apparent external bulge of the pons; the thick red lines mark approximately the postulated ‘pontine’ boundaries, though there is some variation between sources (compare text Fig. 10 in Swanson 1998; text Fig. 0 in Paxinos and Franklin 2007; see also Dong and The Allen Institute for Brain Science 2008). **c** Schema illustrating present results, ascribing 25 paramedian serotonergic populations to discrete neuromeric origins. The limits of the midbrain (*M*), developmental hindbrain (*H*) units containing the basilar pons (pons proper) and the spinal cord (*SC*) are marked in *red*. Laterally displaced cell groups are not represented in this panel

The serotonergic phenotype is associated to a single paramedian progenitor domain along the hindbrain tagma, which is subdivided into 12 segmental portions (isthmus, r1–r11; Fig. 15c), and seems complemented by neighboring minor domains in the caudal midbrain and rostral spinal cord (it remains unclear whether intrinsic patterning or tangential migrations are involved in these additions). We also found that r4 produces a small number of serotonergic neurons, instead of being a gap rhombomere, unable to produce serotonergic neurons, as is usually thought. There is evidence that, irrespective of the common neurotransmitter phenotype, the cells produced at each segmental level variously reflect the local molecular context (rhombomeric identity) in terms of specific gene expression patterns or gradiental expression patterns, varying layering behaviors (superficial, intermediate or periventricular sites), lateral dispersion behaviors, cell typology, and specific projection patterns. Individual rhombomeres may reproduce or not the overall pattern found in their immediate neighbors, sometimes forming plurineuromeric complexes. Under this light, the classic raphe nuclei can be understood as plurineuromeric complexes, whose cytoarchitectonic definition resulted from lumping together cell groups showing a similar histogenetic pattern consecutively in a few adjacent rhombomeres (for instance DR across m2, Is and r1, RMg, across r5 and r6, or RPa and ROb across r7–r11). This raises the issue whether individual rhombomeric components of such complex anatomic units have shared connectivity and functional properties, or display segmental differences due to local molecular singularities. The literature already contains some data suggesting that the latter case is true, but more research done with this possibility in mind is needed. In this sense, our updated terminology, which adds a segmental reference code for the individual parts, should help in producing more precise descriptions of observed hodologic differences and other differential properties. A similar analysis obviously applies to other hindbrain ‘columnar’ nuclei (Marín and Puelles 1995; Cambronero and Puelles 2000; Marín et al. 2008).

## Abbreviations

4v: Fourth ventricle
5C: Motor trigeminal nucleus, caudal part
5n: Trigeminal nerve
5R: Motor trigeminal nucleus, rostral part
7n: Facial nerve
8n: Vestibulocochlear nerve
10n: Vagus nerve
11n: Accessory nerve
Amb: Ambiguus nucleus
Cb: Cerebellum
CLi: Caudal linear nucleus of the raphe
CLiW: Caudal linear nucleus of the raphe, lateral wing
Cu: Cuneate nucleus
DR: Dorsal raphe nucleus
DTg: Dorsal tegmental nucleus
ECu: External cuneate nucleus
Gu: Gustatory nucleus
Gr: Gracile nucleus
H: Hindbrain
ICo: Inferior colliculus
III: Oculomotor nucleus
IO: Inferior olivary nucleus
IPA: Interpeduncular nucleus, apical subnucleus
IPC: Interpeduncular nucleus, caudal subnucleus
IPPro: Interpeduncular nucleus, prodromal subnucleus
IPR: Interpeduncular nucleus, rostral subnucleus
Is: Isthmus
isDR: Isthmic part of dorsal raphe nucleus
isDRd: Isthmic part of dorsal raphe nucleus, dorsal part
isDRl: Isthmic part of dorsal raphe nucleus, lateral part
isDRv: Isthmic part of dorsal raphe nucleus, ventral part
isDRW: Isthmic part of dorsal raphe nucleus, lateral wing
IV: Trochlear nucleus
LC: Locus coeruleus
LLV: Ventral nucleus of the lateral lemniscus
LRt: Lateral reticular nucleus
LVe: Lateral vestibular nucleus
M: Midbrain
m2: Mesomere 2 (preisthmic midbrain)
mDR: Dorsal raphe nucleus, preisthmic mesencephalic part
mesV: Mesencephalic trigeminal nucleus
MHB: Midbrain–hindbrain boundary
mlf: Medial longitudinal fasciculus
MnR: Median raphe nucleus
MnRc: Median raphe nucleus, caudal part
MnRr: Median raphe nucleus, rostral part
MVe: Medial vestibular nucleus
my: Myelomere
PB: Parabigeminal nucleus
PBr: Parabrachial nucleus
PDTg: Posterodorsal tegmental nucleus
PMB: Pontomedullary boundary
Pn: Pontine nuclei
PnR: Pontine raphe nucleus
PO: Periolivary area
PPnR: Prepontine raphe nucleus
PPy: Parapyramidal raphe complex
Pr5C: Principal sensory trigeminal nucleus, caudal part
Pr5R: Principal sensory trigeminal nucleus, rostral part
py: Pyramidal tract
r1: Rhombomere 1
r1c: Caudal part of rhombomere 1
r1DR: r1 part of dorsal raphe nucleus
r1DRd: r1 part of dorsal raphe nucleus, dorsal part
r1DRv: r1 part of dorsal raphe nucleus, ventral part
r1DRW: r1 part of dorsal raphe nucleus, lateral wing
r1r: Rostral part of rhombomere 1
r2: Rhombomere 2
r3: Rhombomere 3
r3PnR: r3 part of pontine raphe nucleus
r4: Rhombomere 4
r4PnR: r4 part of pontine raphe nucleus
r5: Rhombomere 5
r5RMgD: r5 part of raphe magnus nucleus, dorsal part
r5RMgV: r5 part of raphe magnus nucleus, ventral part
r5SGeR: r5 part of supragenual raphe nucleus
r6: Rhombomere 6
r6RMgD: r6 part of raphe magnus nucleus, dorsal part
r6RMgV: r6 part of raphe magnus nucleus, ventral part
r6SGeR: r6 part of supragenual raphe nucleus
r7: Rhombomere 7
r8: Rhombomere 8
r9: Rhombomere 9
r10: Rhombomere 10
r11: Rhombomere 11
R: Red nucleus
Rbd: Rhabdoid nucleus
RMg: Raphe magnus nucleus
RMgD: Raphe magnus nucleus, dorsal part
RMgV: Raphe magnus nucleus, ventral part
ROb: Raphe obscurus nucleus
RPa: Raphe pallidus nucleus
RtTg: Reticular tegmental nucleus of the pons
Sag: Nucleus sagulum
SC: Spinal cord
SCo: Superior colliculus
SGeR: Supragenual raphe nucleus
SO: Superior olive
Sol: Nucleus of the solitary tract
Sp5C: Spinal trigeminal nucleus, caudal part
Sp5I: Spinal trigeminal nucleus, interpolar part
Sp5O: Spinal trigeminal nucleus, oral part
SpVe: Spinal vestibular nucleus
SuL: Supralemniscal raphe complex
Tz: Trapezoid body
Ve: Vestibular nucleus
VI: Abducens nucleus
VII: Facial nucleus
X: Vagal dorsal motor nucleus
XI: Accessory nucleus
XII: Hypoglossal nucleus
xpn: Pontine decussation
xpy: Pyramidal decussation
xscp: Decussation of the superior cerebellar peduncle
xtz: Trapezoid body decussation
